# Global, regional, and national burden of multiple sclerosis 1990–2016: a systematic analysis for the Global Burden of Disease Study 2016

**DOI:** 10.1016/S1474-4422(18)30443-5

**Published:** 2019-03

**Authors:** GBD 2016 Multiple Sclerosis Collaborators, Mitchell T Wallin, William J Culpepper, Emma Nichols, Zulfiqar A Bhutta, Tsegaye Tewelde Gebrehiwot, Simon I Hay, Ibrahim A Khalil, Kristopher J Krohn, Xiaofeng Liang, Mohsen Naghavi, Ali H Mokdad, Molly R Nixon, Robert C Reiner, Benn Sartorius, Mari Smith, Roman Topor-Madry, Andrea Werdecker, Theo Vos, Valery L. Feigin, Christopher J L Murray

## Abstract

**Background:**

Multiple sclerosis is the most common inflammatory neurological disease in young adults. The Global Burden of Diseases, Injuries, and Risk Factors Study (GBD) provides a systematic method of quantifying various effects of a given condition by demographic variables and geography. In this systematic analysis, we quantified the global burden of multiple sclerosis and its relationship with country development level.

**Methods:**

We assessed the epidemiology of multiple sclerosis from 1990 to 2016. Epidemiological outcomes for multiple sclerosis were modelled with DisMod-MR version 2.1, a Bayesian meta-regression framework widely used in GBD epidemiological modelling. Assessment of multiple sclerosis as the cause of death was based on 13 110 site-years of vital registration data analysed in the GBD's cause of death ensemble modelling module, which is designed to choose the optimum combination of mathematical models and predictive covariates based on out-of-sample predictive validity testing. Data on prevalence and deaths are summarised in the indicator, disability-adjusted life-years (DALYs), which was calculated as the sum of years of life lost (YLLs) and years of life lived with a disability. We used the Socio-demographic Index, a composite indicator of income per person, years of education, and fertility, to assess relations with development level.

**Findings:**

In 2016, there were 2 221 188 prevalent cases of multiple sclerosis (95% uncertainty interval [UI] 2 033 866–2 436 858) globally, which corresponded to a 10·4% (9·1 to 11·8) increase in the age-standardised prevalence since 1990. The highest age-standardised multiple sclerosis prevalence estimates per 100 000 population were in high-income North America (164·6, 95% UI, 153·2 to 177·1), western Europe (127·0, 115·4 to 139·6), and Australasia (91·1, 81·5 to 101·7), and the lowest were in eastern sub-Saharan Africa (3·3, 2·9–3·8), central sub-Saharan African (2·8, 2·4 to 3·1), and Oceania (2·0, 1·71 to 2·29). There were 18 932 deaths due to multiple sclerosis (95% UI 16 577 to 21 033) and 1 151 478 DALYs (968 605 to 1 345 776) due to multiple sclerosis in 2016. Globally, age-standardised death rates decreased significantly (change −11·5%, 95% UI −35·4 to −4·7), whereas the change in age-standardised DALYs was not significant (−4·2%, −16·4 to 0·8). YLLs due to premature death were greatest in the sixth decade of life (22·05, 95% UI 19·08 to 25·34). Changes in age-standardised DALYs assessed with the Socio-demographic Index between 1990 and 2016 were variable.

**Interpretation:**

Multiple sclerosis is not common but is a potentially severe cause of neurological disability throughout adult life. Prevalence has increased substantially in many regions since 1990. These findings will be useful for resource allocation and planning in health services. Many regions worldwide have few or no epidemiological data on multiple sclerosis, and more studies are needed to make more accurate estimates.

**Funding:**

Bill & Melinda Gates Foundation.

## Introduction

Multiple sclerosis is the most common inflammatory neurological disease in young adults. The mean age of diagnosis is approximately 30 years, with most patients presenting with periodic neurological relapses.^1^ One to two decades after onset, many patients with multiple sclerosis enter a progressive phase of the disease. Several environmental factors and genetic alleles might alter the risk of developing multiple sclerosis, but the underlying cause of the disease remains elusive.^2^, ^3^ Common neurological manifestations of multiple sclerosis include optic neuritis, diplopia, sensory loss, limb weakness, gait ataxia, loss of bladder control, and cognitive dysfunction. 15 medications have been approved by the US Food and Drug Administration to reduce the number of relapses and attenuate progression of neurological disability.^1^ These drugs are partly effective, but whether they alter the long-term course of multiple sclerosis remains unclear.^4^

The epidemiology of multiple sclerosis has been the subject of many studies. Over the past five decades, prevalence has been rising across North America and Europe, high incidence has been seen among women and among African Americans, and persistent geographical risk gradients have been documented.^5^, ^6^, ^7^, ^8^, ^9^ Contemporary reviews have provided updates on the prevalence, incidence, and burden of multiple sclerosis in the Americas,^5^ Europe,^6^ and worldwide.^7^ The MS International Federation has compiled a useful international atlas of the epidemiology of multiple sclerosis with detailed country-level data, but this group did not use systematic modelling for disease morbidity, mortality, and risk factors.^10^ The lack of standardisation in the methods of epidemiological studies limits comparisons of findings and assessments of multiple sclerosis with those of other conditions. Additionally, few evaluations have been done at the country level, epidemiological data in different ethnic groups are incomplete, and many regions of the world remain to be studied.

**Research in context**

**Evidence before this study**

We searched PubMed for studies published between 2011 and 2015 that were representative, population-based surveys, including retrospective case-report and hospital-report analyses and nationally representative health studies. The search string used was “multiple sclerosis AND epidemiology AND (2011/01/01: 2015/12/31)”. Additional searches for features such as prevalence, disability-adjusted life-years, years of life lost, and mortality are reviewed in the appendix. Global estimates of prevalence, incidence, and deaths from multiple sclerosis have been made in past iterations of the Global Burden of Diseases, Injuries, and Risk Factors Study (GBD). Other epidemiological studies have focused on variations in prevalence and incidence of multiple sclerosis between individual countries and noted marked differences along latitude gradients. Similar data have been recorded on a map produced by the MS International Federation. The GBD study uses an integrated modelling approach to predict morbidity and mortality in countries and regions of the world from where there are insufficient data. Previously, estimates for multiple sclerosis have been summarised in GBD reports about other diseases, but no report has focused exclusively on this disease.

**Added value of this study**

This Article presents the methods and results for a focused GBD 2016 analysis of multiple sclerosis, making use of detailed country-level morbidity and mortality data. We have explored variation in the burden of multiple sclerosis by country development level, as measured by the Socio-demographic Index, which is a composite indicator of income per person, years of education, and fertility.

**Implications of all the available evidence**

Multiple sclerosis is quite rare but is a potentially severe cause of neurological disability. Onset is typically in young adulthood, after which symptoms persist throughout adult life. Prevalence has increased in many world regions, due in part to improved survival. These findings are relevant to researchers, clinicians, and health policy makers. The health service needs of the increasing number of patients with multiple sclerosis must be considered. Several disease-modifying therapies have been developed that reduce the number of relapses and slow neurological disability, at least during the early phases of the disease. Although expensive, these medications are important components of comprehensive multiple sclerosis care, along with rehabilitation and access to multidisciplinary care. More national, epidemiological studies in multiple sclerosis are needed for GBD to generate more robust worldwide estimates in the future.

The Global Burden of Diseases, Injuries, and Risk Factors Study (GBD) provides a systematic method of quantifying health loss in great detail for a given condition by demographic variables and geographical location.^11^ By using information from the literature, population statistics, and rigorous algorithms, a consistent picture is produced across the major regions of the world. Comparisons of multiple sclerosis with other common neurological conditions were reported by the GBD 2015 Neurological Disorders Collaborator Group.^12^ In 2015, multiple sclerosis ranked tenth for prevalence among neurological conditions measured, with 2 012 000 cases estimated globally.^12^ Prevalence of multiple sclerosis and disability-adjusted life-years (DALYs) were significantly higher in women than in men, and there were significant gradients in prevalence and incidence across different regions of the world.

In this systematic analysis, we present new estimates of the global burden of multiple sclerosis from 1990 to 2016 (measured by prevalence, mortality, DALYs, years of life lived with disability [YLDs], and years of life lost [YLLs]), and assess the relation with development level, as measured by the Socio-demographic Index (SDI). The GBD approach provides a perspective on the burden of multiple sclerosis relevant to researchers, clinicians, and health policy makers.

## Methods

The general methods of GBD 2016 are summarised in the appendix, and we present here the methods specific to the estimation of multiple sclerosis. This study complies with the Guidelines for Accurate and Transparent Health Estimates Reporting (GATHER) recommendations. Further details on GBD 2016 data, analysis, and results are available online.

### Mortality

The International Classification of Diseases code for multiple sclerosis is 340 in the ninth edition and G35 in the tenth edition. Modelling of causes of death related to multiple sclerosis was based on 13 110 site-years of data (ie, a combination of GBD locations and calendar years of vital registration data) and analysed in the GBD cause of death ensemble modelling tool (CODEm). The model used predictive covariates, including population-weighted average latitude by country, mean cholesterol, the GDB Healthcare Access and Quality Index, the cumulative number of cigarettes consumed over the previous 5 and 10 years, education, lag-distributed income, and SDI (a composite indicator of income per person, years of education, and fertility; appendix). The choice of covariates was based on any reported putative relationship with multiple sclerosis mortality, but does not imply any causal relationships. CODEm is designed to assess which combination of covariates best helps to fit a model to the available input data by use of out-of-sample predictive validity testing.

### Modelling of prevalence and incidence

The reference case definition of multiple sclerosis for GBD is based on the 2005 McDonald criteria.^13^ We also incorporated data with alternative definitions, including the Poser criteria,^14^ Schumacher's criteria,^15^ Allison and Millar criteria,^16^ Bauer criteria,^17^ and McAlpine criteria (appendix).^18^ Other sources reporting on medical claims data using International Classification of Diseases codes or general practice codes from the UK were also used. From a systematic review, 167 unique sources informing the epidemiological model for multiple sclerosis were identified. We added US medical claims data from 3 years (2000, 2010, and 2012). We found 129 unique sources on prevalence and 65 unique sources on incidence, covering 13 of the 21 GBD world regions (appendix). No data were available for southeast Asia, Oceania, eastern Europe, the Caribbean, Andean Latin America, central sub-Saharan Africa, southern sub-Saharan Africa, and western sub-Saharan Africa. We used the GATHER checklist to define reporting practices for studies, like the GBD study, that calculate health estimates for multiple populations using multiple data sources (appendix).^19^

Epidemiological outcomes for multiple sclerosis were modelled with DisMod-MR 2.1, a Bayesian meta-regression framework widely used in GBD epidemiological modelling. This framework combines data on prevalence, incidence, remission, and mortality into one model. We set incidence to zero among children younger than 4 years and assumed no remission (ie, no full cure). Study covariates were included to adjust the US claims data for 2000 and 2010, which we found to be systematically lower than claims data for 2012. SDI was included as a covariate on prevalence, average latitude as a covariate on prevalence and incidence, and the GBD Healthcare Access and Quality Index as a covariate on excess mortality (ie, excess deaths in people with multiple sclerosis compared with mortality in people without multiple sclerosis; appendix).

### Severity distributions and years lived with disability

We identified four separate health states of multiple sclerosis based on disability level: mild, moderate, severe, and asymptomatic. The latter category represents people who have a diagnosis of multiple sclerosis but no overt symptoms. In the GBD Disability Weight Surveys,^20^ short lay descriptions were written to capture the main symptoms, reported according to Kurtzke's Expanded Disability Status Scale^21^ and assuming that scores of 0 indicate asymptomatic disease, scores greater than 0 up to 3·5 indicate mild multiple sclerosis, those greater than 3·5 and up to 6·5 indicate moderate multiple sclerosis, and scores greater than 6·5 indicate severe disease (appendix).^21^ A systematic review of studies with severity data according to the Expanded Disability Status Scale identified 27 unique sources, covering ten of 21 GBD world regions. However, only 14 of these studies separated people with scores of 0, whereas the other studies included scores of 0 in the mild category. To make use of all available data on severity, a two-step meta-analytical approach was developed. First, we did a meta-analysis of studies with data on the proportion of people with asymptomatic multiple sclerosis to determine the proportions of those with asymptomatic or mild multiple sclerosis included in the overall mild category. We did the second meta-analysis on all available data on the proportion of the total mild category (asymptomatic and mild combined) and the proportions of moderate and severe multiple sclerosis. Finally, the proportions of asymptomatic and mild from the first meta-analysis were scaled to the total mild category that was generated in the second meta-analysis to estimate the final proportions for the four health state categories. The prevalence estimates for each severity state were multiplied by the corresponding disability weights to derive uncorrected YLDs. These initial YLD estimates were subsequently corrected for comorbidity with the comorbidity correction simulation developed for GBD.^22^

### Risk estimation

Relative risk data were pooled with a meta-analysis of cohort, case-control, and intervention studies. Risks and outcomes were paired, and for each we evaluated the evidence and judged whether the evidence fell into the categories of convincing or probable, as defined by the World Cancer Research Fund.^23^ From the prevalence and relative risk results, population-attributable fractions were estimated relative to the theoretical minimum risk exposure level. When we aggregated estimates for clusters of risks, such as metabolic or behavioural risks, we used a multiplicative function rather than simple addition, taking into account how much of each risk is mediated through another risk. The choice of covariates was based on any reported putative relationship with multiple sclerosis morbidity, but does not imply any causal relationships.

Smoking was the only environmental risk of 84 risks quantified in GBD 2016 that we judged to have sufficient evidence for a causal relationship with multiple sclerosis as an outcome.^24^ Population-based surveys were the main source of data for the smoking exposure model. Population-attributable fractions were estimated based on these exposure data, along with data on relative risk and a theoretical minimum level of exposure, which for smoking was no lifetime exposure to tobacco smoke. Criteria for inclusion of risks into GBD include the availability of sufficient evidence for a causal relationship between a risk and one or more disease or injury outcome; evidence to support generalisability of an effect size beyond the populations included in epidemiological studies; availability of sufficient data and methods to enable estimation of exposure levels by country; and the likely importance of a risk factor to disease burden or policy considerations. Additional details on risk factor calculations have been described elsewhere.^25^

### Compilation of results

YLLs were calculated by multiplying the number of deaths in each age group by the remaining life expectancy for that age group, as determined by the GBD standard life table.^26^ DALYs were calculated as the sum of YLLs and YLDs. Uncertainty was propagated through all calculations by sampling 1000 draws at each step of the calculations, carrying through uncertainty from input data, corrections of measurement error, and estimates of residual non-sampling error. Uncertainty intervals (UIs) were defined as the 25th and 975th values of the ordered draws. The significance of changes between 1990 and 2016 was based on the 95% UI of the change estimate, not including zero.

### Role of the funding source

The funder of the study had no role in study design, data collection, data analysis, data interpretation or the writing of the report. The corresponding authors had full access to all the data in the study and had final responsibility for the decision to submit for publication.

## Results

**Figure 1:**
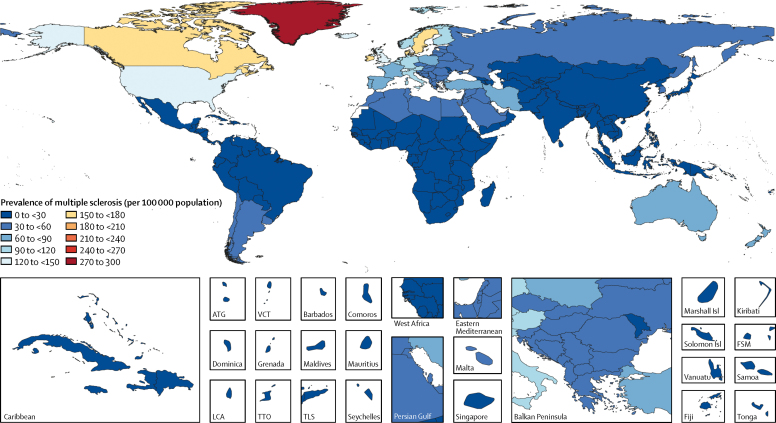
Age-standardised multiple sclerosis prevalence per 100 000 population in 2016 for both sexes, by location ATG=Antigua and Barbuda. Isl=Islands. LCA=Saint Lucia. VCT=Saint Vincent and the Grenadines. TTO=Trinidad and Tobago. TLS=Timor-Leste. FSM=Federated States of Micornesia.

In 2016, an estimated 2 221 188 people worldwide had multiple sclerosis (95% UI 2 033 866–2 436 858), corresponding to a prevalence of 30·1 cases (95% UI 27·5–33·0) per 100 000 population. Age-standardised prevalence estimates increased by 22·47 cases (95% UI 20·5–24·61) per 100 000 population or 10·4% (9·1–11·8) between 1990 and 2016 (table). Age-standardised prevalence was greater than 120 cases per 100 000 population in North America and some northern European countries, moderate (60–120 per 100 000) in some countries in Europe and Australasia, and lowest (<60 per 100 000 population) in North Africa and the Middle East, Latin America, Asia, Oceania, the Caribbean, and sub-Saharan Africa (figure 1). Of note, in low prevalence regions, many countries had no data and modelling was used to generate estimates (appendix). Detailed estimates on morbidity and mortality by year (1990–2016) and geographical region can be found on the GBD 2016 Global Health Data Exchange. Age-standardised prevalence of multiple sclerosis changed most in the east Asia region (44·8% increase) and Canada (81·9% increase) between 1990 and 2016. The highest age-standardised multiple sclerosis prevalence estimates per 100 000 population were in high-income North America (164·6, 95% UI, 153·2–177·1), western Europe (127·0, 115·4–139·6), and Australasia (91·1, 81·5–101·7), and the lowest were in eastern sub-Saharan Africa (3·3, 2·9–3·8), central sub-Saharan Africa (2·8, 2·4–3·1), and Oceania (2·0, 1·71–2·29)

**Table:** Deaths, prevalence, and DALYs for multiple sclerosis in 2016, and percentage change of age-standardised rates by location

		**Deaths (95% uncertainty interval)**		**Prevalence (95% uncertainty interval)**		**DALYs (95% uncertainty interval)**	
		2016 counts	Percentage change in age-standardised rates between 1990 and 2016 (%)	2016 counts	Percentage change in age-standardised rates between 1990 and 2016 (%)	2016 counts	Percentage change in age-standardised rates between 1990 and 2016 (%)
**Global**		**18 932 (16 577 to 21 033)**	**−11·5% (−35·4 to −4·7)**	**2 221 188 (2 033 866 to 2 436 858)**	**10·4% (9·1 to 11·8)**	**1 151 478 (968 605 to 1 345 776)**	**−4·2% (−16·4 to 0·8)**
Low SDI		495 (330 to 676)	495 (330 to 676)	41 903 (37 144 to 47 358)	23·0% (21·4 to 24·9)	30 296 (23 152 to 38 812)	12·9% (−5·8 to 31·0)
Low-middle SDI		2433 (2082 to 2975)	6·1% (−18·9 to 56·2)	263 919 (234 904 to 299 835)	33·1% (31·8 to 34·3)	158 748 (132 266 to 189 475)	14·1% (−6·9 to 41·3)
Middle SDI		2884 (2608 to 3422)	0·7% (−19·7 to 34·0)	255 163 (228 209 to 287 299)	41·3% (39·8 to 42·9)	166 937 (142 543 to 193 030)	14·4% (−4·0 to 34·5)
High-middle SDI		3092 (2578 to 4152)	−28·6% (−40·2 to −7·0)	448 405 (404 845 to 498 848)	18·7% (16·5 to 21·1)	222 002 (180 033 to 270 242)	−10·6% (−20·1 to 3·1)
High SDI		10 021 (7385 to 10 873)	2·9% (−34·2 to 12·1)	1 227 486 (1 134 117 to 1 330 378)	30·6% (28·5 to 32·8)	577 314 (473 251 to 678 019)	13·7% (−2·2 to 19·4)
**High income**							
High-income North America		4373 (3124 to 4755)	28·7% (−22·1 to 47·8)	591 642 (550 379 to 636 457)	32·6% (28·7 to 37·0)	264 997 (216 143 to 310 734)	25·7% (7·8 to 32·9)
	Canada	510 (389 to 584)	12·8% (−31·9 to 33·3)	79 419 (71 959 to 86 444)	81·9% (65·5 to 95·9)	33 692 (27 195 to 40 545)	44·1% (21·0 to 61·2)
	Greenland	1 (1 to 2)	−10·2% (−41·6 to 28·6)	162 (147 to 178)	15·6% (11·3 to 20·1)	82 (64 to 106)	−1·9% (−22·9 to 18·2)
	USA	3862 (2743 to 4226)	30·7% (−20·8 to 51·1)	511 855 (477 414 to 549 489)	27·4% (23·1 to 32·6)	231 170 (188 833 to 270 151)	23·4% (6·5 to 31·0)
Australasia		194 (142 to 226)	3·8% (−40·8 to 19·7)	26 106 (23 353 to 29 152)	36·6% (29·6 to 44·7)	11 785 (9540 to 14 271)	19·3% (−3·4 to 33·1)
	Australia	157 (116 to 187)	6·3% (−40·2 to 26·3)	22 298 (19 861 to 25 004)	38·7% (30·6 to 48·2)	9816 (7938 to 11 957)	21·9% (−1·5 to 37·7)
	New Zealand	37 (26 to 45)	−5·5% (−43·2 to 14·2)	3809 (3440 to 4237)	25·7% (20·1 to 31·3)	1969 (1582 to 2360)	7·6% (−14·3 to 22·3)
High-income Asia Pacific		225 (191 to 298)	−25·1% (−39·5 to 2·0)	66 573 (59 014 to 75 430)	17·0% (15·7 to 18·6)	24 124 (18 360 to 30 467)	1·9% (−6·0 to 11·7)
	Brunei	0 (0 to 0)	−1·5% (−25·9 to 29·5)	45 (39 to 52)	33·7% (28·8 to 39·1)	23 (17 to 32)	13·0% (−4·7 to 31·9)
	Japan	167 (141 to 229)	−23·6% (−36·0 to 8·3)	46 249 (41 130 to 52 322)	12·6% (11·6 to 13·7)	16 830 (12 816 to 21 428)	0·0% (−7·2 to 11·5)
	Singapore	4 (3 to 6)	−26·2% (−55·2 to 21·3)	453 (396 to 521)	30·3% (25·4 to 35·8)	258 (203 to 322)	−6·5% (−28·4 to 24·2)
	South Korea	54 (38 to 73)	−28·1% (−51·1 to 5·7)	19 826 (17 449 to 22 482)	31·2% (26·8 to 36·2)	7012 (5247 to 8990)	8·3% (−7·3 to 27·0)
Western Europe		4795 (3451 to 5482)	−2·1% (−40·2 to 8·6)	543 862 (493 933 to 597 684)	26·1% (23·3 to 28·7)	262 909 (214 047 to 309 869)	8·9% (−8·7 to 16·9)
	Andorra	1 (1 to 1)	−0·5% (−31·1 to 44·1)	96 (86 to 107)	17·1% (11·7 to 23·0)	50 (36 to 64)	5·3% (−14·1 to 29·9)
	Austria	97 (65 to 113)	11·4% (−42·8 to 36·1)	10 999 (9896 to 12 169)	30·4% (25·3 to 35·8)	5370 (4281 to 6389)	17·7% (−11·4 to 33·1)
	Belgium	120 (86 to 143)	−7·8% (−33·9 to 10·7)	14 752 (13 171 to 16 352)	20·3% (15·1 to 25·5)	6740 (5433 to 8101)	4·9% (−8·9 to 18·0)
	Cyprus	5 (5 to 6)	−15·3% (−32·9 to 0·9)	660 (585 to 740)	31·9% (24·4 to 39·7)	318 (262 to 380)	5·6% (−9·5 to 21·4)
	Denmark	112 (80 to 136)	−15·4% (−43·1 to 9·4)	11 673 (10 619 to 12 844)	18·5% (12·2 to 25·5)	5812 (4707 to 6949)	−3·3% (−19·5 to 13·8)
	Finland	59 (37 to 71)	6·5% (−47·3 to 37·2)	8209 (7379 to 9075)	22·8% (0·3 to 30·6)	3629 (2801 to 4409)	13·3% (−13·8 to 30·8)
	France	553 (378 to 641)	3·3% (−33·8 to 19·3)	65 467 (58 412 to 72 742)	26·2% (20·6 to 32·1)	31 167 (24 904 to 37 142)	12·9% (−3·8 to 25·4)
	Germany	1165 (884 to 1410)	−7·3% (−39·4 to 9·5)	111 970 (100 711 to 123 187)	21·4% (15·5 to 26·9)	57 865 (47 568 to 69 286)	2·5% (−14·9 to 16·7)
	Greece	81 (56 to 93)	47·2% (−27·3 to 88·6)	7727 (6861 to 8649)	33·2% (28·1 to 39·8)	4375 (3265 to 5267)	40·2% (3·6 to 65·5)
	Iceland	4 (3 to 5)	8·9% (−39·3 to 32·5)	532 (481 to 585)	38·8% (29·9 to 47·6)	252 (202 to 302)	21·5% (−5·4 to 38·5)
	Ireland	52 (38 to 70)	−14·5% (−46·3 to 18·7)	8054 (7221 to 8867)	26·1% (20·1 to 32·9)	3536 (2806 to 4336)	4·9% (−12·1 to 23·4)
	Israel	24 (15 to 30)	13·0% (−42·9 to 51·2)	4077 (3626 to 4593)	13·3% (8·4 to 19·3)	1763 (1336 to 2208)	13·1% (−11·0 to 33·9)
	Italy	398 (280 to 475)	−2·0% (−40·0 to 14·5)	72 352 (64 659 to 80 555)	31·7% (23·2 to 40·6)	29 059 (22 643 to 35 453)	14·7% (−1·1 to 29·8)
	Luxembourg	7 (5 to 9)	−13·1% (−49·5 to 7·0)	845 (763 to 931)	18·5% (13·7 to 23·9)	406 (323 to 489)	0·5% (−20·7 to 14·6)
	Malta	2 (1 to 3)	−5·8% (−43·9 to 22·4)	228 (202 to 257)	21·3% (17·5 to 25·9)	111 (86 to 134)	8·2% (−14·1 to 26·5)
	Netherlands	219 (169 to 262)	−13·1% (−35·8 to 8·4)	25 197 (22 660 to 27 789)	16·7% (11·4 to 22·0)	12 190 (9 815 to 14 529)	0·6% (−12·2 to 14·5)
	Norway	88 (54 to 108)	6·0% (−42·2 to 28·8)	7518 (6827 to 8250)	7·7% (−2·0 to 17·7)	4224 (3282 to 5046)	3·9% (−18·6 to 18·8)
	Portugal	50 (39 to 82)	−16·5% (−41·8 to 17·8)	8367 (7425 to 9439)	29·0% (24·0 to 34·5)	3500 (2655 to 4586)	5·9% (−10·9 to 25·1)
	Spain	215 (166 to 280)	1·1% (−41·1 to 18·6)	43 867 (39 811 to 48 085)	47·3% (27·5 to 56·9)	17 272 (13 654 to 21 085)	24·9% (4·7 to 40·9)
	Sweden	126 (83 to 150)	7·2% (−37·9 to 31·7)	20 304 (18 607 to 21 890)	50·9% (43·0 to 59·7)	8138 (6499 to 9791)	27·2% (5·4 to 40·3)
	Switzerland	127 (82 to 173)	−16·7% (−45·0 to 15·0)	13 968 (12 552 to 15 522)	22·1% (12·2 to 32·4)	6658 (5088 to 8463)	−0·2% (−18·3 to 18·4)
	UK	1290 (922 to 1413)	8·1% (−38·0 to 19·0)	106 454 (97 402 to 115 773)	28·5% (25·9 to 31·3)	60 333 (49 758 to 69 626)	12·4% (−11·2 to 19·6)
Southern Latin America		156 (129 to 220)	−20·0% (−36·3 to 16·0)	31 209 (27 321 to 35 483)	29·0% (22·7 to 37·5)	12 908 (10 106 to 16 120)	4·0% (−9·7 to 24·5)
	Argentina	113 (91 to 168)	−18·2% (−35·4 to 22·7)	20 248 (17 490 to 23 365)	28·0% (18·6 to 40·6)	8734 (6745 to 11 090)	3·2% (−12·7 to 29·1)
	Chile	27 (19 to 38)	−9·4% (−45·7 to 31·0)	9362 (8242 to 10 622)	34·0% (28·6 to 39·7)	3249 (2410 to 4194)	17·3% (−3·3 to 38·2)
	Uruguay	17 (13 to 19)	−9·6% (−28·4 to 15·9)	1598 (1396 to 1793)	18·3% (12·9 to 23·3)	924 (754 to 1109)	0·6% (−12·4 to 18·8)
**Central Europe, eastern Europe, and central Asia**							
Eastern Europe		1284 (907 to 1913)	−15·3% (−39·8 to 24·5)	126 031 (111 553 to 142 447)	26·2% (22·2 to 30·8)	79 274 (61 518 to 104 707)	−1·9% (−21·0 to 27·0)
	Belarus	49 (27 to 93)	−9·4% (−44·7 to 26·0)	4832 (4280 to 5445)	27·5% (22·6 to 32·6)	2932 (2007 to 4599)	2·9% (−20·3 to 26·9)
	Estonia	10 (7 to 13)	−29·5% (−48·2 to 9·1)	913 (810 to 1016)	20·7% (14·7 to 26·3)	573 (442 to 697)	−17·7% (−32·9 to 11·4)
	Latvia	20 (14 to 24)	−16·8% (−35·7 to 14·8)	1303 (1168 to 1451)	19·3% (13·9 to 24·7)	1004 (767 to 1200)	−9·5% (−24·2 to 16·3)
	Lithuania	29 (21 to 34)	−10·8% (−25·8 to 15·8)	1852 (1645 to 2062)	22·2% (16·4 to 27·8)	1436 (1171 to 1663)	−3·7% (−17·3 to 19·4)
	Moldova	10 (8 to 13)	6·1% (−26·3 to 28·8)	1335 (1170 to 1518)	27·4% (23·1 to 33·6)	736 (596 to 899)	14·9% (−8·7 to 32·8)
	Russia	754 (480 to 1259)	−21·9% (−51·4 to 38·6)	93 975 (83 041 to 106 775)	27·6% (22·8 to 33·6)	51 657 (38 194 to 71 187)	−4·2% (−27·9 to 36·6)
	Ukraine	413 (270 to 595)	2·1 (−30·2 to 39·2)	21 821 (19 266 to 24 501)	18·3% (13·5 to 23·3)	20 937 (15 211 to 27 804)	6·2% (−20·0 to 37·5)
Central Europe		1042 (901 to 1307)	−25·9% (−38·8 to 14·5)	78 560 (69 822 to 88 256)	32·5% (26·9 to 43·1)	51 263 (43 136 to 60 647)	−13·4% (−25·9 to 20·0)
	Albania	35 (25 to 55)	−28·0% (−50·7 to 34·8)	1727 (1397 to 2008)	37·4% (30·7 to 46·6)	1280 (870 to 2176)	−5·6% (−26·3 to 35·5)
	Bosnia and Herzegovina	28 (21 to 47)	−14·9% (−34·9 to 24·7)	1748 (1550 to 1975)	32·0% (26·2 to 38·0)	1329 (1066 to 1869)	−3·7% (−20·7 to 24·3)
	Bulgaria	66 (47 to 116)	−1·8% (−24·0 to 21·5)	3115 (2749 to 3529)	20·1% (14·9 to 25·6)	2587 (1864 to 4233)	8·1% (−13·0 to 27·9)
	Croatia	40 (29 to 48)	2·1% (−38·3 to 26·7)	2019 (1792 to 2262)	25·3% (19·6 to 30·7)	1677 (1318 to 1970)	5·4% (−23·7 to 24·3)
	Czech Republic	98 (78 to 126)	−41·9% (−55·4 to 4·8)	7382 (6596 to 8216)	6·2% (0·3 to 12·6)	4682 (3837 to 5776)	−30·8% (−43·7 to 4·2)
	Hungary	87 (66 to 102)	−24·6% (−41·7 to 15·5)	6681 (5927 to 7480)	21·2% (14·7 to 28·7)	4290 (3506 to 5008)	−14·1% (−28·0 to 17·9)
	Macedonia	14 (12 to 17)	5·9% (−11·6 to 24·2)	939 (826 to 1 059)	31·7% (26·3 to 36·9)	670 (575 to 786)	11·7% (−2·2 to 26·3)
	Montenegro	5 (4 to 6)	−7·2% (−22·4 to 9·8)	305 (270 to 340)	18·9% (14·0 to 24·0)	243 (207 to 286)	−2·1% (−15·2 to 12·1)
	Poland	418 (330 to 547)	−35·0% (−51·7 to 23·9)	36 049 (31 697 to 42 604)	38·4% (28·1 to 63·3)	21 855 (17 916 to 26 607)	−20·4% (−37·5 to 29·1)
	Romania	95 (75 to 143)	−41·8% (−57·5 to 18·5)	8433 (7444 to 9571)	32·3% (25·8 to 41·2)	5242 (4181 to 6670)	−26·9% (−43·0 to 23·2)
	Serbia	92 (75 to 105)	10·8% (−13·1 to 32·3)	5304 (4748 to 5945)	27·9% (23·0 to 33·3)	4307 (3467 to 5001)	15·0% (−1·7 to 32·2)
	Slovakia	39 (26 to 48)	17·9% (−31·1 to 57·2)	3372 (3002 to 3790)	33·5% (28·4 to 38·6)	2052 (1566 to 2486)	20·8% (−11·0 to 45·1)
	Slovenia	25 (16 to 31)	−19·8% (−52·2 to 4·8)	1487 (1318 to 1663)	10·7% (4·4 to 17·6)	1048 (808 to 1253)	−12·6% (−36·0 to 6·8)
Central Asia		120 (90 to 181)	−28·9% (−47·6 to 13·6)	19 521 (17 022 to 22 350)	26·5% (23·7 to 29·2)	9192 (7082 to 11 849)	−1·9% (−14·0 to 17·8)
	Armenia	8 (6 to 9)	18·7% (−23·9 to 44·6)	801 (702 to 919)	39·7% (33·7 to 46·5)	460 (354 to 562)	24·2% (−2·0 to 41·1)
	Azerbaijan	10 (8 to 13)	−7·8% (−29·0 to 18·8)	2309 (2004 to 2667)	32·3% (27·3 to 37·6)	995 (771 to 1 241)	13·1% (−0·8 to 29·3)
	Georgia	8 (5 to 10)	32·3% (−18·7 to 72·6)	1097 (959 to 1240)	22·7% (18·0 to 28·2)	503 (379 to 624)	20·7% (1·8 to 40·1)
	Kazakhstan	47 (28 to 88)	−17·5% (−44·5 to 19·9)	5347 (4648 to 6161)	26·6% (20·6 to 32·5)	2876 (1984 to 4397)	3·4% (−15·5 to 24·2)
	Kyrgyzstan	4 (4 to 5)	−35·7% (−50·8 to 9·3)	1017 (883 to 1179)	20·9% (16·3 to 25·5)	442 (340 to 551)	−10·9% (−25·7 to 18·0)
	Mongolia	4 (3 to 6)	−35·4% (−55·6 to 37·6)	714 (618 to 827)	36·7% (31·5 to 42·2)	330 (249 to 436)	−8·4% (−30·4 to 37·8)
	Tajikistan	5 (4 to 7)	−16·8% (−37·5 to 11·5)	1112 (967 to 1279)	24·6% (19·8 to 30·1)	508 (399 to 629)	3·7% (−10·3 to 19·0)
	Turkmenistan	11 (7 to 15)	1·0% (−35·4 to 50·9)	1197 (1035 to 1385)	49·9% (43·3 to 56·7)	644 (467 to 873)	22·5% (−3·4 to 48·1)
	Uzbekistan	23 (17 to 41)	−60·2% (−75·8 to 68·7)	5927 (5152 to 6851)	35·8% (29·4 to 42·2)	2434 (1801 to 3205)	−13·6% (−34·3 to 48·6)
**Latin America and Caribbean**							
Central Latin America		463 (328 to 515)	39·8% (−25·3 to 63·1)	20 566 (18 080 to 23 437)	40·7% (39·0 to 42·7)	21 437 (16 674 to 24 408)	37·3% (−12·7 to 53·3)
	Colombia	68 (38 to 82)	17·5% (−44·7 to 44·7)	2662 (2312 to 3056)	33·4% (28·8 to 39·2)	2982 (2052 to 3592)	16·5% (−32·6 to 37·0)
	Costa Rica	8 (6 to 10)	0·3% (−25·6 to 35·4)	355 (312 to 405)	29·4% (23·7 to 35·1)	355 (288 to 441)	8·1% (−11·5 to 36·5)
	El Salvador	6 (5 to 8)	2·2% (−15·3 to 55·5)	402 (353 to 460)	42·8% (37·5 to 48·6)	323 (268 to 406)	12·1% (−3·3 to 52·6)
	Guatemala	15 (9 to 20)	35·9% (−26·8 to 92·2)	846 (742 to 966)	42·3% (37·3 to 47·8)	766 (563 to 977)	34·4% (−11·6 to 74·8)
	Honduras	11 (7 to 18)	17·8% (−23·0 to 79·1)	450 (392 to 516)	36·6% (31·8 to 41·9)	517 (355 to 776)	19·4% (−16·4 to 68·2)
	Mexico	286 (196 to 316)	56·6% (−22·8 to 88·2)	13 353 (11 799 to 15 169)	43·9% (41·8 to 45·9)	13 484 (10 243 to 15 282)	48·9% (−10·7 to 69·7)
	Nicaragua	7 (5 to 9)	29·6% (−4·1 to 68·8)	346 (301 to 398)	33·2% (28·9 to 38·6)	339 (278 to 405)	28·0% (3·8 to 55·1)
	Panama	6 (5 to 8)	11·8% (−26·3 to 39·0)	255 (224 to 294)	29·5% (24·0 to 34·2)	284 (219 to 343)	14·3% (−12·9 to 38·0)
	Venezuela	55 (38 to 70)	19·8% (−20·7 to 56·4)	1897 (1651 to 2179)	32·5% (27·5 to 37·7)	2387 (1833 to 2972)	23·4% (−6·3 to 55·3)
Andean Latin America		61 (51 to 71)	1·5% (−25·8 to 20·0)	3772 (3308 to 4352)	31·1% (27·8 to 34·4)	2976 (2505 to 3531)	5·7% (−13·9 to 20·2)
	Bolivia	15 (11 to 19)	0·7% (−28·3 to 35·8)	797 (698 to 914)	36·3% (31·5 to 40·8)	688 (542 to 843)	6·0% (−19·6 to 35·3)
	Ecuador	17 (13 to 20)	−3·9% (−34·4 to 23·5)	816 (708 to 943)	31·1% (25·7 to 36·8)	785 (644 to 926)	2·5% (−20·1 to 26·8)
	Peru	29 (23 to 37)	5·9% (−23·3 to 37·4)	2159 (1891 to 2487)	30·3% (25·5 to 34·9)	1503 (1196 to 1895)	8·1% (−12·0 to 27·7)
Caribbean		101 (83 to 118)	5·0% (−24·9 to 18·1)	5307 (4689 to 6001)	22·1% (20·0 to 24·2)	4817 (4080 to 5661)	5·9% (−16·8 to 15·7)
	Antigua and Barbuda	0 (0 to 0)	−24·2% (−46·9 to 26·0)	12 (11 to 14)	10·0% (3·7 to 18·1)	12 (9 to 17)	−17·7% (−37·5 to 22·8)
	The Bahamas	1 (1 to 1)	−29·7% (−45·8 to 12·7)	64 (56 to 72)	13·2% (8·5 to 17·4)	53 (45 to 63)	−21·1% (−35·9 to 12·4)
	Barbados	1 (1 to 1)	0·6% (−17·9 to 22·1)	39 (33 to 44)	13·3% (6·7 to 18·7)	49 (41 to 60)	4·9% (−11·3 to 23·5)
	Belize	0 (0 to 0)	31·4% (−10·9 to 130·1)	28 (25 to 33)	34·0% (29·4 to 38·7)	20 (16 to 24)	30·0% (0·2 to 85·3)
	Bermuda	0 (0 to 0)	−48·4% (−66·1 to 12·7)	15 (13 to 17)	15·5% (11·3 to 19·9)	8 (6 to 12)	−30·6% (−46·9 to 10·4)
	Cuba	45 (32 to 52)	11·9% (−30·3 to 33·6)	1988 (1760 to 2237)	21·5% (17·7 to 26·3)	1930 (1509 to 2249)	8·8% (−22·0 to 24·2)
	Dominica	0 (0 to 0)	−13·5% (−34·9 to 42·5)	7 (7 to 9)	27·4% (22·1 to 33·1)	5 (4 to 6)	1·2% (−16·5 to 37·1)
	Dominican Republic	11 (9 to 13)	2·0% (−18·7 to 29·8)	1031 (907 to 1180)	34·5% (29·8 to 38·8)	644 (517 to 787)	10·5% (−5·1 to 28·1)
	Grenada	0 (0 to 1)	−27·6% (−59·8 to 114·8)	10 (9 to 12)	14·0% (4·1 to 31·6)	16 (12 to 24)	−26·4% (−56·6 to 92·1)
	Guyana	1 (1 to 1)	0·0% (−25·8 to 41·1)	42 (36 to 48)	26·9% (21·5 to 31·8)	41 (33 to 53)	8·9% (−12·7 to 39·2)
	Haiti	18 (11 to 32)	−1·2% (−32·7 to 36·6)	709 (624 to 809)	25·6% (21·4 to 30·6)	883 (607 to 1 484)	1·9% (−29·9 to 34·8)
	Jamaica	4 (3 to 5)	0·6% (−25·9 to 58·7)	294 (260 to 337)	21·0% (16·9 to 25·3)	205 (162 to 268)	6·9% (−13·1 to 44·2)
	Puerto Rico	16 (10 to 19)	26·5% (−28·9 to 54·0)	625 (550 to 711)	28·8% (23·6 to 33·6)	676 (477 to 809)	24·7% (−17·7 to 46·3)
	Saint Lucia	0 (0 to 0)	−12·7% (−31·9 to 30·5)	18 (16 to 21)	29·2% (23·7 to 34·4)	15 (12 to 19)	−4·0% (−20·4 to 29·3)
	Saint Vincent and the Grenadines	0 (0 to 0)	−26·5% (−45·8 to 57·8)	9 (8 to 11)	24·1% (19·8 to 28·6)	7 (6 to 10)	−16·0% (−33·9 to 46·1)
	Suriname	1 (1 to 1)	−0·6% (−18·4 to 50·0)	34 (30 to 39)	26·5% (21·7 to 31·8)	34 (28 to 44)	3·7% (−11·2 to 40·4)
	Trinidad and Tobago	2 (2 to 3)	7·9% (−26·4 to 25·6)	134 (118 to 155)	25·5% (20·1 to 30·4)	120 (97 to 141)	16·5% (−10·8 to 31·3)
	Virgin Islands	1 (1 to 1)	−4·9% (−31·9 to 25·3)	23 (18 to 30)	25·8% (8·9 to 61·0)	39 (30 to 49)	−3·9% (−32·5 to 25·7)
Tropical Latin America		382 (327 to 502)	9·4% (−31·6 to 29·7)	30 337 (26 678 to 34 681)	35·7% (33·2 to 38·2)	19 304 (16 128 to 23 108)	19·2% (−10·7 to 30·1)
	Brazil	375 (321 to 496)	8·6% (−32·4 to 29·1)	29 467 (25 915 to 33 687)	35·7% (33·2 to 38·2)	18 835 (15 729 to 22 660)	18·8% (−11·2 to 29·8)
	Paraguay	7 (4 to 9)	42·9% (0·4 to 128·5)	870 (764 to 992)	32·6% (27·8 to 38·0)	469 (343 to 580)	35·2% (15·1 to 69·1)
**Southeast Asia, east Asia, and Oceania**							
East Asia		1286 (1129 to 1492)	−22·4% (−42·4 to 3·4)	106 397 (94 021 to 119 349)	44·8% (42·9 to 46·8)	72 483 (62 038 to 83 775)	−4·7% (−26·8 to 14·9)
	China	1230 (1 076 to 1 422)	−22·9% (−43·3 to 3·6)	103 194 (91 242 to 115 717)	45·6% (43·6 to 47·6)	69 708 (59 718 to 80 673)	−4·9% (−27·4 to 15·2)
	North Korea	29 (19 to 45)	9·1% (−8·8 to 28·9)	1703 (1501 to 1926)	10·3% (6·8 to 13·9)	1507 (1107 to 2062)	9·8% (−2·9 to 24·5)
	Taiwan (province of China)	27 (22 to 34)	−23·9% (−41·9 to −3·9)	1501 (1309 to 1713)	49·5% (37·5 to 69·8)	1267 (1056 to 1510)	−7·3% (−24·4 to 10·8)
Southeast Asia		579 (509 to 766)	−5·3% (−27·0 to 42·8)	22 495 (19 644 to 25 753)	36·7% (34·4 to 39·1)	26 642 (23 053 to 33 448)	0·9% (−20·2 to 37·7)
	Cambodia	13 (10 to 18)	0·7% (−31·8 to 54·1)	415 (359 to 478)	42·7% (36·7 to 49·0)	596 (482 to 774)	4·8% (−27·1 to 49·3)
	Indonesia	187 (156 to 274)	11·5% (−12·7 to 54·6)	7056 (6124 to 8123)	37·1% (34·4 to 40·1)	8754 (7323 to 11 847)	13·6% (−9·0 to 45·6)
	Laos	5 (4 to 7)	−1·1% (−38·0 to 41·5)	207 (180 to 237)	41·6% (36·1 to 46·8)	254 (198 to 326)	4·7% (−31·8 to 38·8)
	Malaysia	23 (19 to 28)	−5·9% (−23·0 to 44·4)	876 (759 to 1006)	41·4% (36·1 to 46·8)	1048 (903 to 1216)	1·6% (−14·4 to 40·9)
	Maldives	0 (0 to 0)	−23·5% (−62·1 to 21·9)	9 (8 to 10)	52·6% (46·6 to 58·7)	8 (5 to 12)	−15·2% (−53·2 to 26·6)
	Mauritius	1 (1 to 2)	−14·6% (−37·2 to 23·9)	78 (68 to 88)	31·0% (26·4 to 36·5)	67 (54 to 80)	−2·7% (−20·4 to 26·4)
	Myanmar	93 (77 to 125)	−11·1% (−46·4 to 43·3)	2458 (2156 to 2805)	44·1% (39·6 to 49·9)	3999 (3332 to 5155)	−8·0% (−43·7 to 37·6)
	Philippines	75 (56 to 118)	−5·9% (−30·5 to 45·8)	2989 (2601 to 3426)	27·8% (23·3 to 32·8)	3530 (2746 to 5087)	−0·8% (−22·5 to 37·6)
	Sri Lanka	19 (14 to 28)	−17·0% (−41·7 to 44·1)	704 (614 to 805)	38·2% (33·1 to 43·1)	822 (642 to 1099)	−8·8% (−30·0 to 38·9)
	Seychelles	0 (0 to 0)	11·0% (−26·7 to 49·7)	4 (3 to 4)	35·3% (24·1 to 43·5)	10 (6 to 14)	8·2% (−25·9 to 45·9)
	Thailand	66 (56 to 83)	−13·7% (−31·6 to 29·8)	3518 (3080 to 3998)	36·6% (31·9 to 41·3)	3166 (2673 to 3811)	−1·2% (−17·0 to 32·0)
	Timor-Leste	1 (0 to 1)	0·5% (−46·2 to 62·6)	22 (19 to 25)	42·1% (37·4 to 47·3)	24 (16 to 34)	5·1% (−39·2 to 55·8)
	Vietnam	95 (70 to 129)	−10·2% (−35·6 to 42·8)	4126 (3595 to 4710)	36·4% (32·0 to 41·5)	4354 (3365 to 5608)	−4·1% (−27·0 to 36·0)
Oceania		7 (4 to 14)	−6·0% (−25·1 to 33·2)	221 (192 to 257)	24·9% (22·0 to 27·9)	329 (203 to 588)	−1·5% (−19·6 to 32·7)
	American Samoa	0 (0 to 0)	−0·4% (−28·4 to 50·7)	3 (3 to 3)	31·3% (26·6 to 35·8)	3 (2 to 5)	7·2% (−16·5 to 49·3)
	Federated States of Micronesia	0 (0 to 0)	8·5% (−29·3 to 85·6)	2 (2 to 2)	39·7% (33·9 to 45·9)	4 (2 to 6)	12·8% (−23·8 to 81·4)
	Fiji	1 (1 to 2)	0·4% (−35·4 to 59·0)	37 (32 to 42)	29·5% (24·8 to 34·5)	50 (27 to 95)	4·0% (−27·5 to 53·9)
	Guam	0 (0 to 0)	3·1% (−22·6 to 35·1)	7 (6 to 8)	22·8% (18·6 to 27·2)	8 (5 to 14)	8·4% (−12·8 to 35·8)
	Kiribati	0 (0 to 0)	7·4% (−15·5 to 56·4)	2 (1 to 2)	32·3% (26·7 to 38·0)	4 (2 to 6)	10·1% (−12·4 to 54·5)
	Marshall Islands	0 (0 to 0)	−3·5% (−30·2 to 41·4)	1 (1 to 2)	29·6% (24·8 to 34·6)	2 (1 to 4)	3·9% (−19·5 to 43·3)
	Northern Mariana Islands	0 (0 to 0)	−5·0% (−29·7 to 28·5)	6 (5 to 6)	27·0% (22·6 to 31·7)	5 (3 to 7)	2·8% (−17·7 to 30·0)
	Papua New Guinea	5 (2 to 9)	−8·7% (−31·3 to 34·1)	122 (105 to 143)	29·8% (24·8 to 35·0)	209 (122 to 407)	−5·1% (−26·8 to 33·4)
	Samoa	0 (0 to 0)	−7·2% (−28·8 to 27·1)	5 (4 to 6)	24·2% (19·3 to 28·9)	6 (5 to 9)	−1·8% (−22·6 to 26·8)
	Solomon Islands	0 (0 to 1)	1·5% (−25·9 to 52·9)	10 (9 to 12)	30·8% (26·2 to 35·9)	19 (11 to 37)	5·9% (−20·9 to 52·3)
	Tonga	0 (0 to 0)	−5·5% (−30·7 to 36·9)	4 (3 to 4)	26·9% (22·1 to 31·5)	4 (3 to 5)	1·0% (−23·3 to 35·4)
	Vanuatu	0 (0 to 0)	3·9% (−23·8 to 59·4)	7 (6 to 8)	32·2% (27·3 to 37·2)	11 (7 to 20)	8·5% (−17·8 to 58·8)
**North Africa and Middle East**							
North Africa and Middle East		936 (801 to 1157)	13·1% (−7·5 to 53·0)	248 843 (223 208 to 277 059)	20·4% (17·3 to 23·6)	100 215 (79 849 to 120 731)	16·1% (5·7 to 29·9)
	Afghanistan	64 (30 to 148)	22·8% (−4·8 to 60·8)	6297 (5593 to 7112)	12·2% (7·8 to 17·1)	4380 (2703 to 8187)	18·2% (−1·8 to 41·2)
	Algeria	58 (48 to 68)	10·6% (−15·3 to 49·2)	15 334 (13 546 to 17 335)	21·5% (17·0 to 26·0)	6164 (4892 to 7694)	15·3% (−2·0 to 35·8)
	Bahrain	1 (1 to 2)	−8·9% (−36·4 to 27·2)	543 (477 to 620)	10·7% (6·5 to 15·1)	197 (149 to 253)	3·0% (−12·9 to 20·7)
	Egypt	84 (63 to 124)	11·3% (−14·3 to 57·4)	29 566 (26 112 to 33 348)	17·0% (12·3 to 21·7)	10 803 (8124 to 13 681)	13·3% (−2·4 to 32·5)
	Iran	304 (240 to 416)	29·7% (−9·5 to 133·8)	58 650 (53 713 to 63 984)	40·4% (28·3 to 51·8)	26 395 (20 990 to 32 645)	35·2% (8·8 to 74·2)
	Iraq	40 (28 to 53)	8·4% (−20·5 to 48·4)	9707 (8594 to 11 040)	3·9% (−0·1 to 9·0)	4205 (3239 to 5219)	5·0% (−10·6 to 25·3)
	Jordan	12 (8 to 17)	−0·3% (−31·4 to 53·7)	3138 (2761 to 3557)	11·5% (6·1 to 16·5)	1324 (972 to 1710)	6·1% (−12·8 to 30·8)
	Kuwait	3 (2 to 5)	−3·8% (−34·8 to 50·1)	2039 (1647 to 2542)	76·2% (53·3 to 110·9)	669 (474 to 924)	45·4% (17·8 to 83·9)
	Lebanon	8 (6 to 10)	−27·1% (−53·8 to 4·6)	3428 (3036 to 3897)	16·4% (11·8 to 21·1)	1164 (882 to 1477)	0·7% (−16·6 to 21·6)
	Libya	11 (8 to 19)	27·6% (−11·0 to 90·3)	2797 (2466 to 3181)	25·5% (20·7 to 30·3)	1170 (877 to 1531)	24·9% (5·0 to 51·8)
	Morocco	52 (43 to 64)	27·5% (−4·9 to 79·5)	14 253 (12 702 to 16 219)	24·7% (18·8 to 30·0)	5591 (4359 to 6863)	23·3% (4·8 to 45·9)
	Oman	4 (4 to 6)	22·2% (−14·8 to 221·6)	1327 (1159 to 1520)	59·1% (53·5 to 65·0)	542 (416 to 681)	39·1% (15·0 to 96·3)
	Palestine	8 (5 to 9)	4·1% (−17·0 to 32·0)	1140 (1009 to 1287)	−2·9% (−6·6 to 1·1)	617 (459 to 758)	−0·7% (−15·4 to 15·1)
	Qatar	3 (2 to 4)	3·8% (−42·6 to 74·0)	862 (759 to 978)	−0·4% (−4·9 to 4·1)	349 (259 to 453)	−1·7% (−23·0 to 21·7)
	Saudi Arabia	24 (20 to 32)	28·6% (−8·8 to 196·3)	10 840 (9572 to 12 346)	65·4% (62·0 to 69·2)	3817 (2934 to 4761)	48·4% (31·7 to 83·1)
	Sudan	32 (23 to 46)	18·8% (−10·3 to 71·2)	6333 (5586 to 7181)	23·0% (18·4 to 29·2)	2989 (2276 to 3824)	19·5% (0·8 to 42·9)
	Syria	21 (18 to 27)	7·4% (−16·5 to 69·4)	6947 (6158 to 7889)	31·2% (26·5 to 36·1)	2568 (1962 to 3220)	20·3% (2·9 to 43·7)
	Tunisia	21 (16 to 25)	15·3% (−13·5 to 65·9)	6063 (5388 to 6880)	24·3% (19·3 to 29·6)	2301 (1781 to 2851)	20·1% (2·1 to 42·0)
	Turkey	147 (106 to 183)	−17·4% (−46·6 to 15·9)	61 408 (54 238 to 69 106)	14·3% (7·7 to 19·6)	21 146 (16 029 to 26 706)	3·0% (−15·0 to 20·6)
	United Arab Emirates	18 (11 to 30)	34·3% (−13·8 to 115·9)	4216 (3655 to 4807)	22·8% (17·6 to 27·8)	1900 (1369 to 2598)	25·6% (1·2 to 58·1)
	Yemen	21 (14 to 34)	23·3% (−10·1 to 115·1)	3674 (3251 to 4193)	29·0% (24·2 to 34·4)	1849 (1379 to 2501)	23·7% (0·2 to 61·5)
**South Asia**							
South Asia		2475 (2128 to 3032)	13·9% (−13·0 to 72·7)	260 957 (232 320 to 295 620)	32·7% (31·4 to 34·0)	159 378 (132 698 to 190 622)	19·4% (−1·6 to 49·2)
	Bangladesh	165 (107 to 217)	−32·0% (−60·4 to −5·2)	22 400 (19 675 to 25 592)	27·4% (22·9 to 31·5)	12 298 (9290 to 15 187)	−11·2% (−40·3 to 11·1)
	Bhutan	1 (1 to 1)	−5·8% (−48·8 to 57·4)	136 (120 to 155)	41·3% (35·5 to 46·2)	77 (60 to 97)	8·4% (−28·8 to 43·6)
	India	2024 (1726 to 2508)	19·0% (−8·5 to 79·7)	204 460 (182 397 to 231 531)	32·6% (31·3 to 34·0)	127 296 (105 717 to 151 997)	22·3% (1·4 to 53·0)
	Nepal	41 (26 to 54)	5·3% (−33·0 to 59·3)	4182 (3709 to 4717)	33·1% (27·8 to 38·3)	2574 (1871 to 3208)	11·6% (−19·6 to 41·4)
	Pakistan	244 (184 to 347)	24·1% (−5·2 to 134·2)	29 780 (26 263 to 33 750)	36·6% (31·8 to 42·0)	17 133 (13 630 to 21 638)	27·4% (5·3 to 72·9)
**Sub-Saharan Africa**							
Southern sub-Saharan Africa		70 (61 to 81)	8·8% (−6·6 to 53·0)	8331 (7365 to 9448)	21·4% (20·0 to 23·0)	4734 (3948 to 5603)	11·0% (1·0 to 31·4)
	Botswana	2 (1 to 3)	9·4% (−55·1 to 111·3)	207 (181 to 237)	28·1% (23·7 to 32·6)	121 (77 to 168)	17·0% (−23·0 to 71·6)
	Lesotho	2 (1 to 3)	26·3% (−24·7 to 172·7)	182 (161 to 209)	25·0% (20·7 to 29·4)	109 (84 to 141)	26·7% (−7·5 to 90·5)
	Namibia	2 (1 to 2)	−9·9% (−49·3 to 40·6)	187 (165 to 213)	30·2% (26·1 to 35·8)	105 (74 to 140)	3·5% (−23·9 to 35·3)
	South Africa	55 (48 to 65)	2·6% (−11·9 to 41·6)	6868 (6071 to 7778)	21·2% (19·7 to 22·8)	3798 (3142 to 4521)	7·3% (−2·9 to 26·3)
	Swaziland	1 (0 to 1)	−2·3% (−41·6 to 63·1)	100 (88 to 115)	17·7% (13·8 to 21·6)	56 (41 to 74)	6·5% (−18·8 to 43·0)
	Zimbabwe	9 (6 to 13)	57·9% (6·9 to 179·5)	787 (690 to 896)	12·3% (8·8 to 16·0)	545 (396 to 707)	36·9% (7·5 to 83·5)
Western sub-Saharan Africa		166 (107 to 242)	−2·8% (−32·1 to 28·8)	14 426 (12 614 to 16 447)	21·7% (19·6 to 24·0)	10 242 (7565 to 13 409)	4·4% (−16·7 to 24·1)
	Benin	6 (3 to 8)	1·0% (−25·1 to 49·7)	378 (330 to 433)	17·7% (13·6 to 21·8)	311 (220 to 415)	6·2% (−13·2 to 36·9)
	Burkina Faso	9 (5 to 13)	−4·8% (−32·4 to 40·6)	592 (514 to 681)	20·6% (16·5 to 25·0)	507 (346 to 661)	2·2% (−22·0 to 31·5)
	Cameroon	12 (8 to 18)	0·7% (−27·9 to 38·1)	751 (650 to 868)	16·7% (12·4 to 21·3)	640 (480 to 876)	5·2% (−15·5 to 32·1)
	Cape Verde	0 (0 to 1)	27·4% (−18·3 to 96·1)	36 (32 to 41)	34·3% (28·4 to 39·4)	25 (16 to 34)	28·0% (1·8 to 64·3)
	Chad	6 (3 to 9)	−7·9% (−34·9 to 24·4)	486 (421 to 555)	15·9% (12·0 to 19·7)	348 (243 to 474)	−1·0% (−21·3 to 20·5)
	Côte d'Ivoire	13 (10 to 19)	6·0% (−14·9 to 55·6)	753 (652 to 867)	23·3% (18·9 to 27·3)	708 (560 to 934)	10·6% (−6·2 to 46·5)
	The Gambia	1 (0 to 1)	−3·1% (−31·1 to 26·0)	72 (63 to 83)	18·8% (14·4 to 23·2)	51 (35 to 68)	2·4% (−16·9 to 22·1)
	Ghana	16 (11 to 22)	16·7% (−12·9 to 54·9)	1144 (1001 to 1317)	26·4% (22·0 to 31·4)	926 (727 to 1 158)	19·3% (−1·9 to 43·2)
	Guinea	6 (4 to 10)	2·9% (−27·2 to 40·2)	429 (373 to 491)	16·2% (12·1 to 20·7)	354 (255 to 470)	6·9% (−15·0 to 33·2)
	Guinea-Bissau	1 (1 to 2)	−6·7% (−35·1 to 32·4)	71 (62 to 81)	17·9% (14·1 to 22·4)	67 (49 to 93)	−1·4% (−24·4 to 27·8)
	Liberia	2 (1 to 3)	4·3% (−22·6 to 40·3)	134 (117 to 154)	17·8% (14·1 to 21·7)	106 (76 to 140)	7·9% (−12·8 to 29·9)
	Mali	7 (3 to 11)	−16·1% (−51·8 to 19·5)	657 (576 to 745)	17·9% (13·2 to 21·9)	454 (296 to 620)	−7·5% (−37·7 to 17·4)
	Mauritania	3 (1 to 4)	−11·5% (−51·7 to 30·4)	258 (226 to 292)	22·6% (19·0 to 26·4)	172 (117 to 234)	−1·3% (−29·7 to 28·4)
	Niger	9 (3 to 14)	−7·6% (−40·5 to 25·3)	673 (591 to 764)	8·8% (5·0 to 12·5)	503 (299 to 702)	−3·2% (−27·0 to 20·1)
	Nigeria	60 (31 to 97)	−9·6% (−49·3 to 26·9)	6901 (6072 to 7898)	23·8% (20·1 to 28·0)	4165 (2883 to 5659)	1·1% (−25·9 to 25·3)
	São Tomé and Príncipe	0 (0 to 0)	3·0% (−24·8 to 55·5)	5 (4 to 6)	22·6% (18·6 to 28·5)	4 (3 to 5)	8·0% (−12·2 to 39·6)
	Senegal	9 (5 to 13)	7·5% (−15·4 to 52·4)	616 (539 to 706)	20·1% (16·0 to 23·8)	503 (356 to 655)	11·1% (−5·8 to 37·6)
	Sierra Leone	3 (2 to 4)	7·4% (−13·6 to 73·8)	212 (185 to 244)	21·4% (17·2 to 26·0)	172 (134 to 227)	11·6% (−4·5 to 54·2)
	Togo	4 (3 to 6)	4·8% (−25·2 to 61·9)	257 (224 to 294)	23·0% (19·2 to 26·8)	226 (169 to 304)	10·3% (−12·0 to 48·1)
Eastern sub-Saharan Africa		163 (104 to 242)	−2·7% (−31·5 to 34·2)	12 793 (11 215 to 14 585)	24·9% (23·0 to 26·9)	9706 (7254 to 12 870)	3·6% (−21·5 to 28·1)
	Burundi	4 (2 to 6)	−17·8% (−48·3 to 19·1)	273 (237 to 314)	14·0% (9·7 to 18·2)	228 (158 to 310)	−11·6% (−40·8 to 18·5)
	Comoros	0 (0 to 1)	−2·8% (−34·3 to 41·5)	35 (31 to 40)	25·5% (21·1 to 30·0)	26 (20 to 36)	4·3% (−20·5 to 33·6)
	Djibouti	1 (0 to 1)	8·8% (−25·1 to 103·9)	44 (39 to 51)	31·0% (26·7 to 35·1)	32 (23 to 45)	14·5% (−9·8 to 63·8)
	Eritrea	3 (2 to 5)	−1·7% (−33·6 to 59·1)	238 (208 to 272)	29·4% (24·7 to 34·7)	184 (139 to 251)	4·9% (−22·5 to 46·5)
	Ethiopia	47 (27 to 72)	−11·6% (−46·2 to 27·9)	3278 (2859 to 3758)	26·4% (21·8 to 31·3)	2665 (1907 to 3619)	−4·3% (−38·0 to 28·0)
	Kenya	15 (10 to 22)	22·7% (−5·4 to 81·5)	1617 (1429 to 1837)	29·5% (27·8 to 31·2)	1013 (779 to 1333)	25·1% (6·2 to 59·2)
	Madagascar	14 (8 to 21)	−1·8% (−27·8 to 33·4)	1277 (1123 to 1451)	18·1% (14·2 to 22·4)	887 (641 to 1 195)	3·9% (−16·3 to 26·5)
	Malawi	7 (4 to 11)	−1·1% (−40·2 to 53·6)	626 (545 to 716)	21·2% (17·0 to 25·3)	437 (309 to 593)	3·5% (−28·4 to 39·4)
	Mozambique	15 (7 to 25)	−10·5% (−41·3 to 27·1)	1184 (1038 to 1353)	22·2% (18·0 to 26·9)	875 (573 to 1 234)	−3·1% (−30·8 to 23·4)
	Rwanda	4 (3 to 7)	−2·8% (−48·4 to 49·4)	333 (290 to 382)	36·8% (31·3 to 42·2)	250 (184 to 344)	6·0% (−35·1 to 44·3)
	Somalia	5 (3 to 8)	−1·3% (−22·0 to 31·7)	277 (240 to 318)	14·7% (10·3 to 19·5)	249 (181 to 392)	2·1% (−16·4 to 26·6)
	South Sudan	4 (2 to 7)	9·3% (−19·4 to 57·3)	340 (294 to 387)	17·2% (12·9 to 20·8)	255 (157 to 376)	10·6% (−11·7 to 37·5)
	Tanzania	24 (16 to 34)	3·0% (−27·4 to 57·0)	1700 (1482 to 1944)	26·1% (22·1 to 30·6)	1357 (1023 to 1796)	8·1% (−16·0 to 43·2)
	Uganda	11 (7 to 17)	−0·5% (−33·1 to 55·2)	883 (768 to 1 023)	30·4% (25·5 to 35·0)	688 (530 to 917)	7·2% (−21·1 to 45·0)
	Zambia	10 (6 to 15)	27·8% (−20·7 to 112·8)	677 (591 to 779)	24·5% (20·3 to 28·7)	556 (405 to 797)	26·4% (−10·5 to 80·6)
Central sub-Saharan Africa		44 (30 to 64)	1·6% (−19·5 to 21·8)	3239 (2814 to 3704)	15·0% (12·4 to 17·5)	2536 (1974 to 3340)	4·6% (−12·5 to 19·2)
	Angola	11 (7 to 17)	10·0% (−32·6 to 73·8)	920 (804 to 1052)	28·2% (24·2 to 32·5)	687 (504 to 932)	14·1% (−21·0 to 56·7)
	Central African Republic	3 (2 to 5)	2·3% (−24·3 to 40·1)	151 (131 to 174)	14·0% (9·8 to 18·2)	135 (93 to 221)	5·7% (−16·1 to 34·0)
	Congo (Brazzaville)	2 (2 to 4)	−5·5% (−37·7 to 40·6)	147 (128 to 169)	24·5% (19·4 to 29·4)	128 (93 to 195)	1·1% (−27·8 to 37·5)
	Democratic Republic of the Congo	26 (16 to 41)	−0·5% (−18·4 to 16·5)	1924 (1671 to 2206)	7·9% (4·6 to 11·2)	1509 (1097 to 2049)	0·9% (−11·9 to 13·2)
	Equatorial Guinea	0 (0 to 1)	−9·4% (−62·1 to 81·9)	33 (29 to 38)	62·7% (56·1 to 69·6)	24 (15 to 38)	5·7% (−42·2 to 71·6)
	Gabon	1 (1 to 2)	5·5% (−27·4 to 55·6)	64 (56 to 74)	24·7% (20·6 to 29·4)	54 (40 to 82)	10·9% (−14·4 to 48·4)

DALY=disability-adjusted life-years. SDI=Socio-demographic Index.

We found a gradient for prevalence from low to high between low and high SDI quintiles. The greatest change in multiple sclerosis prevalence between 1990 and 2016 was seen in the middle SDI quintile, with an increase in prevalence of 41·3% (changes in other quintiles were increases of 30·6% in the high quintile, 18·7% in the high-middle quintile, 33·1% in the low-middle quintile, and 23·0% in the low quintile). We found a significant association between prevalence and latitude. The coefficient of average latitude in our DisMod-MR model was 1·03 (95% UI 1·03–1·04) per degree of latitude which translates to an almost nine times difference in prevalence between countries at the equator and the highest population-weighted average latitude of 74·7°.

**Figure 2:**
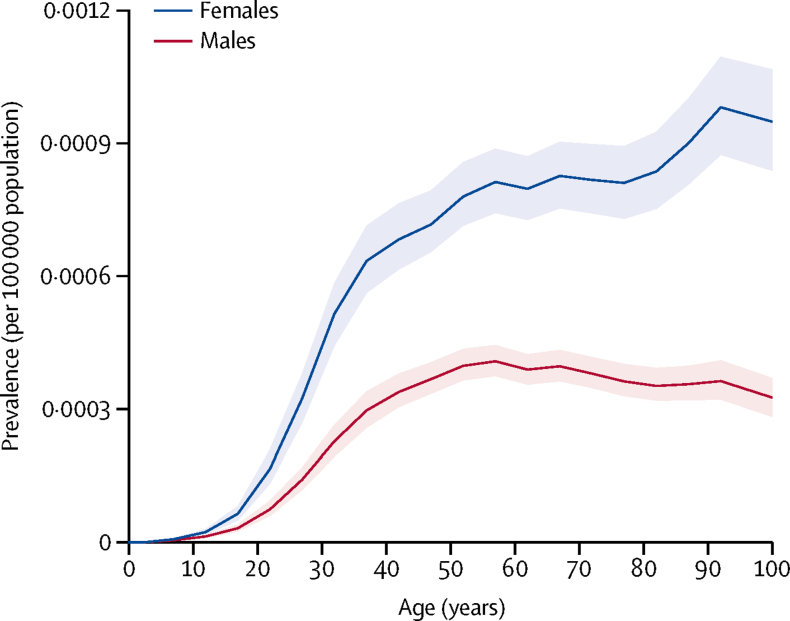
Age-standardised prevalence of multiple sclerosis in 2016, by age and sex Shading shows 95% uncertainty intervals.

The global prevalence of multiple sclerosis differs substantially by sex (figure 2). Among preteen children, the prevalence of multiple sclerosis is similar in boys and girls. During adolescence, the curves start to diverge, with the prevalence increasing more among girls than boys. This pattern continues until around the end of the sixth decade of life, when the sex ratio is 2:1 in favour of women. In older people, prevalence generally continues to climb for women, but a slow attenuation in prevalence is seen for men.

Globally, there were 18 932 deaths due to multiple sclerosis (95% UI 16 577–21 033) in 2016 (table). Most deaths occurred in high SDI countries (10 021 95% UI 7358–10 873), and mortality in the USA was highest among all countries (3862 deaths, 2743–4226). Between 1990 and 2016, the age-standardised mortality rate for multiple sclerosis globally decreased by 11·5%, but changes by region and country were mostly not significant because of wide uncertainty intervals (table).

**Figure 3:**
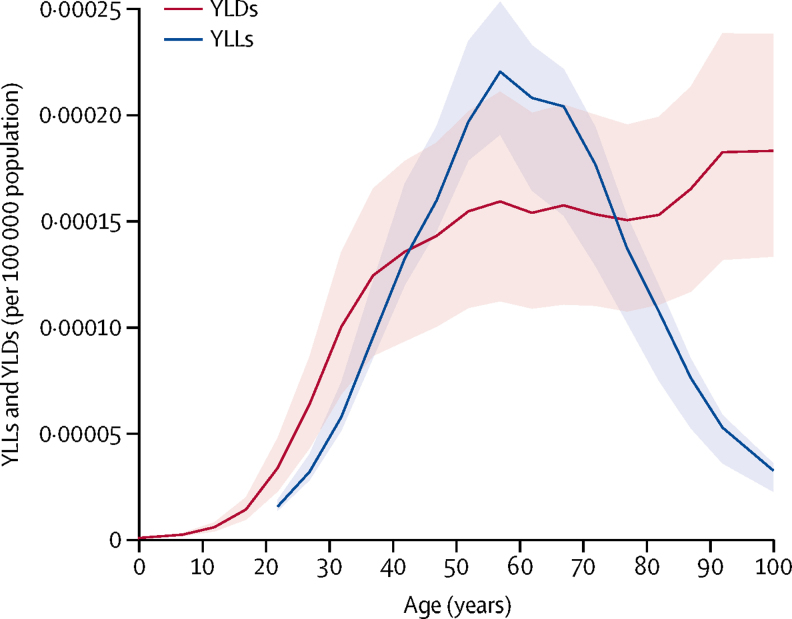
YLDs and YLLs due to multiple sclerosis in 2016, by age Shading shows 95% uncertainty intervals. YLDs=years lived with disability. YLLs=years of life lost.

The effect on YLLs due to premature death and disability was greatest in the sixth decade of life (22·05, 95% UI 19·08–25·34) rising steeply beforehand and dropping substantially afterwards (figure 3). For YLDs, the curve rises to a peak at age 55 years, stabilises, then climbs slightly higher during the eighth decade of life and more steeply thereafter (figure 3).

The global DALYs for multiple sclerosis in 2016 totalled 1 151 478 (95% UI 968 605 to 1 345 776) representing a non-significant decrease of 4·2% (95% UI −16·4 to 0·8) from 1990 (table). Countries in the SDI highest SDI quintile accounted for half of the DALYs in 2016 (577 314 DALYs [50%] of 1 151 478 worldwide). Overall, multiple sclerosis made up 0·04% (95% UK 0·04 to 0·05) of the overall DALYs from all neurological disorders, as shown on the GBD 2016 website.^27^

**Figure 4:**
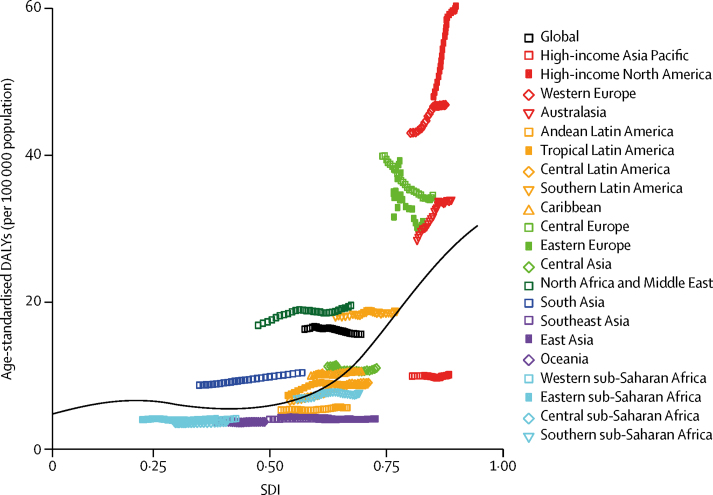
Age-standardised DALYs for multiple sclerosis by SDI, 1990–2016, and expected value-based SDI The black line represents the average expected relationship between SDI and DALYs for multiple sclerosis based on values from all countries over the 1990–2016 estimation period. DALYs= disability-adjusted life-years. SDI=Socio-demographic Index.

The relationship between age-standardised DALYs and SDI over time for each of the 21 GBD regions, represented as annual time series from 1990 and 2016, are shown in figure 4. Age-standardised DALYs rose substantially over time for high-income North America, western Europe, and Australasia. By contrast, in central Europe and eastern Europe age-adjusted DALYs declined while SDI increased. However, among all changes only the increase in high-income North America was significant (table). Among high-income regions, high-income Asia Pacific had DALYs that were much closer to those of low-income and middle-income regions.

Of the global DALYs due to multiple sclerosis, 99 076 (95% UI 62 164–140 650) or 8·6% (95% UI 5·6–11·7) were estimated to be due to smoking.

## Discussion

With the rigorous standardised GBD approach for morbidity assessments, we estimated that there were around 2·2 million cases of multiple sclerosis worldwide in 2016. In that year, prevalence was 10·4% higher than in 1990. Multiple sclerosis also contributed 0·04% (95% UI 0·04–0·05) of total DALYs and 0·05% (0·04–0·07) of all YLLs in 2016. Although neurological disability progression is variable, DALYs peaked in the sixth decade of life. Because onset is most frequently in early adulthood and because survival has been improved, people with multiple sclerosis are affected throughout adult life, leading to the high number of YLDs. The disability weights for multiple sclerosis are generally high (appendix) and YLDs begin to increase steeply early in the second decade of life.

We found a strong latitude gradient for the prevalence of multiple sclerosis, with an increase in prevalence of 1·03 times per degree of latitude. A north to south decrease in prevalence by latitude gradient has been recognised in North America and western Europe,^28^, ^29^ and a reverse south to north increase in gradient has been reported in Australia.^28^ Temporal trends in prevalence show that these gradients were weakening in the 20th century,^9^ but the distribution of multiple sclerosis can still be generally described as having three zones of frequency or risk (figure 1), as originally proposed by Kurtzke.^30^ In 2016, northern European countries and North America made up the high-risk prevalence zone, with estimates of 100 or more cases of multiple sclerosis per 100 000 population. These regions are bounded by areas of medium frequency (prevalence 30–100 cases per 100 000). Low-frequency areas are centred around the equator, and the prevalence in Asia is less than 30 cases per 100 000 population. Geographical location before onset of multiple sclerosis remains a risk factor for acquisition.^28^ Clear gradients from low to high prevalence between low and high SDI quintiles are reported on the GBD 2016 website. The burden of multiple sclerosis was greatest in the regions with the highest socioeconomic status. These gradients point to environmental risk factors that modulate risk based on location.

Prevalence of multiple sclerosis differed substantially over the adult life of women and men. Rising multiple sclerosis morbidity among women in the later 20th century has been reported in studies of multiple sclerosis prevalence and incidence.^7^, ^30^ An analysis of data from the Danish Multiple Sclerosis Registry showed that incidence in women doubled between 1950 and 2009, whereas increases among men have been more modest.^31^ By contrast, excess mortality among patients with multiple sclerosis in Denmark has declined since 1950.^32^ Environmental changes that might be contributing to the rapid change in incidence among women include the rise in obesity, increased cigarette smoking, and changes in the frequency of breastfeeding infants.^31^, ^33^

Our results are consistent with some previous reports of multiple sclerosis morbidity from around the world. Canadian studies have indicated the highest multiple sclerosis prevalence estimates so far. In the province of British Columbia, prevalence was 179·9 cases per 100 000 population in 2008.^34^ This value is close to our estimate for all of Canada, which fell into the band of 150–180 cases per 100 000 population. However, an estimate of 266·9 cases per 100 000 population in Nova Scotia from 2010^35^ is substantially higher. The prevalence of multiple sclerosis reported from European regions has been variable, with values being higher in northern than in southern regions. For example, national prevalence of 154·5 cases per 100 000 was reported in Denmark in 2005,^36^ and an estimate of 230·6 cases per 100 000 population in Northern Ireland was reported in 2008.^37^ A meta-analysis suggests prevalence below 100 cases per 100 000 population in France and southern and eastern Europe.^6^ Studies of multiple sclerosis prevalence in South America have largely been done in small regions, but a study from Panama produced a national crude prevalence of 5·2 per 100 000,^38^ which is notably lower than the estimates for North America. Studies in Africa have been sparse and those from Asia have had variable quality.^39^ Nevertheless, our world map of multiple sclerosis prevalence is similar to the 2013 MS International Federation world atlas for multiple sclerosis morbidity by country and region.^10^

Incidence of multiple sclerosis has been relatively stable or slightly increased over the past four to five decades in white populations, but has been higher in selected racial groups.^7^, ^8^, ^9^ Therefore, the rising prevalence estimates for multiple sclerosis across high-income regions and countries might mostly reflect improved survival.^40^ In addition, the diagnostic criteria for multiple sclerosis have evolved and earlier diagnosis is possible with the use of neuroimaging, and these factors are likely to be contributing to the increased prevalence observed.^41^

Genetic susceptibility to multiple sclerosis is an important factor that influences risk for onset.^3^ Multiple sclerosis is considered to be a complex genetic disease, with over 200 alleles having been discovered to contribute small risk effects.^3^ Yet the rapid changes by sex, race, and ethnicity in incidence and prevalence over the past few generations give support to environmental factors as drivers of susceptibility to multiple sclerosis.^9^ Prime candidates include infection, such as with the Epstein-Barr virus or other organisms, as the initiator of multiple sclerosis,^42^ with the suggestion that infections start in the gut and spread to the CNS.^42^, ^43^ Environmental exposures, such as smoking,^44^ lack of sunlight,^45^ diet,^46^ changes in the gut microbiome,^47^ and obesity,^48^ have also received support as risk factors for onset of multiple sclerosis.

The GBD 2016 neurological data related to multiple sclerosis provide a pathway for priority setting and service planning in health care in relation to other disorders. The rising cost of multiple sclerosis disease-modifying medications is a major global concern.^49^ Ensuring access to disease-modifying medications as well as rehabilitation and multidisciplinary care will help to slow disability progression and support independence in daily activities for patients with multiple sclerosis. Ageing of the large multiple sclerosis population in North America and Europe will be important for health-care providers and policy makers to assess patients' future health-service needs.^50^ Ways to provide adaptive work environments and cost-effective nursing care options to patients will be important for policy planners in regions where prevalence is increasing.

The GBD methods have limitations for the epidemiological assessment of multiple sclerosis. First, data are absent or extremely sparse for many regions of the world, including Latin America, sub-Saharan Africa, and Asia. As such, the models we used to predict prevalence, DALYs, and YLLs might lead to unusual changes in segments of the data. For example, the rapid increases in prevalence in women older than 80 years and the YLD curve we estimated should be viewed with caution because they are not typical of individual population-based studies. We cannot exclude that the relatively low burden of multiple sclerosis in less-developed countries was related to the underdiagnosis of the condition due to limited access to specialised medical care, imaging resources, and laboratory investigations. Additionally, even in high-income regions where multiple sclerosis is well studied, there are few national prevalence and incidence studies, and case-ascertainment infrastructures are limited. For example, in Greenland our estimate for multiple sclerosis prevalence was based on one study in a small community of fewer than 2000 inhabitants, in which no cases of multiple sclerosis were found between 1950 and 1974.^51^ The uncertainty around this mean incidence value of 0 per 100 000 incidence is so wide that it is compatible with a high prevalence, as predicted by our latitude covariate. We acknowledge that with additional epidemiological data this high estimate for Greenland might be altered, but until such information becomes available we maintain that the results from our model are valid.

A second major limitation was the lack of robust predictive covariates for multiple sclerosis to aid in population-based risk assessments,^24^ which was due partly to the limited pool of longitudinal neurological disability data that is representative of the multiple sclerosis population. We found a significant relationships between multiple sclerosis, prevalence, latitude, level of development. However, by including both covariates we might have underestimated the effect of latitude because of collinearity between SDI and distance from the equator. Inclusion of SDI as a covariate might also have spuriously led to increasing estimates of prevalence over time. Latitude remains a proxy and, therefore, an uncertain predictor for multiple sclerosis as long as there is no established biological basis for the relationship. With sparse data and uncertain predictive covariates, we cannot exclude that some of the variation measured in prevalence is due to measurement error.

One way forward for the assessment of the global burden of multiple sclerosis is to use a validated algorithm approach to estimate prevalence and incidence in population-based health care administrative datasets.^52^ This approach has been successfully applied to North American multiple sclerosis populations.^40^, ^52^ The availability of medical claims data from the USA makes us more confident of the estimates for that country. GBD is actively seeking access to medical claims data in other countries to improve the accuracy of estimates for diseases such as multiple sclerosis, for which every patient can be expected to be in contact with the health-care system if there are no major barriers to accessing care. Through our network of collaborators, we expect future iterations of GBD to be able to add such sources from other countries.

In summary, multiple sclerosis is an important cause of neurological disability throughout adult life. This report gives an integrated, contemporary understanding of the global multiple sclerosis disease burden. Prevalence has increased partly due to improved survival. The GBD approach to estimating multiple sclerosis morbidity and mortality is novel and can be repeated with relative efficiency. Our findings will be useful for resource allocation and health services planning for the growing numbers of patients with multiple sclerosis in ageing societies. More national multiple sclerosis epidemiological studies, especially from low-income and middle-income countries, are needed for the GBD Multiple Sclerosis collaborators to generate robust worldwide estimates in the future.
