# Supplementary material for: Global, regional, and national burden of multiple sclerosis 1990–2016: a systematic analysis for the Global Burden of Disease Study 2016
Source: Lancet Neurol. 2019 Mar;18(3):269–85. doi: 10.1016/S1474-4422(18)30443-5 (PMC6372756; doi:10.1016/S1474-4422(18)30443-5)
Supplement: Supplementary appendix [file mmc1.pdf]

THE LANCET
Neurology

Supplementary appendix

This appendix formed part of the original submission and has been peer reviewed. We post it as supplied by the authors.

Supplement to: GBD 2016 Multiple Sclerosis Collaborators. Global, regional, and national burden of multiple sclerosis 1990–2016: a systematic analysis for the Global Burden of Disease Study 2016. *Lancet Neurol* 2019; published online Jan 21. http://dx.doi.org/10.1016/S1474-4422(18)30443-5.

Web Appendix Material

Global, regional, and national burden of multiple sclerosis 1990–2016:

A systematic analysis for the Global Burden of Disease Study 2016

Summary of General Global Burden of Disease Study Methods

The Institute of Health Metrics and Evaluation with a growing collaboration of scientists produces annual updates of the Global Burden of Disease study. Estimates span the period from 1990 to the most recent completed year. By the time of the release of GBD 2016 in September 2017, there were over 2,700 collaborators in 132 countries who contributed to this global public good. Annual updates allow incorporation of new data and method improvements to ensure that the most up-to-date information is available to policy makers in a timely fashion to help make resource allocation decisions. In this analysis, we have aggregated results from GBD 2016 for 15 disease and injury outcomes that are generally cared for by neurological services. These include infectious conditions (tetanus, meningitis, encephalitis), stroke, brain and other nervous system cancers, traumatic brain injury and spinal cord lesion which are classified outside the more narrowly defined category of neurological disorders in GBD (i.e., Alzheimer disease and other dementias, Parkinson disease, multiple sclerosis, motor neuron disease, idiopathic epilepsy, migraine, tension-type headache and a rest category of less common other neurological disorders). Compared to a previous analysis based on GBD 2015,[1] we were able to add the non-fatal outcomes of traumatic brain injury and spinal cord lesion and medication overuse headache is no longer included as a separate cause but quantified as a consequence of the underlying headache types.

In the methods section of this GBD 2016 Multiple Sclerosis (MS) paper we present a summary of the general methods of the global burden of disease. In the accompanying disease-specific papers we concentrate on methods that are specific to each disorder. The guiding principle of GBD is to assess health loss due to mortality and disability comprehensively where we define disability as any departure from full health. In GBD2016, estimates were made for 195 countries and territories, and 579 subnational locations, for 27 years starting from 1990, for 23 age groups and both sexes. Deaths were estimated for 264 disease and injuries while prevalence and incidence were estimated for 328 diseases and injuries. In order to allow meaningful comparisons between deaths and non-fatal disease outcomes as well as between diseases, the data on deaths and prevalence are summarized in a single indicator, the disability-adjusted life year (DALY). DALYs are the sum of years of life lost (YLLs) and years lived with disability (YLDs). YLLs are estimated as the multiplication of counts of death and a standard, ‘ideal’, remaining life expectancy at the age of death. The standard life expectancy is derived from the lowest observed mortality rates in any population in the world greater than 5 million.[2] YLDs are estimated as the product of prevalence of individual consequences of disease (or ‘sequelae’) times a disability weight that quantifies the relative severity of a sequela as a number between zero (representing ‘full health’) and 1 (representing death). Disability weights have been estimated in nine population surveys and an open-access internet survey in which respondents are asked to choose the ‘healthier’[3] between random pairs of health states that are presented with a short description of the main features.

All-cause mortality rates are estimated from vital registration data in countries with complete coverage. For other countries, the probabilities of death before age 5 and between ages 15 and 60 are estimated from censuses and surveys asking mothers to provide a history of children ever born and those still alive, and surveys asking adults about siblings who are alive or have passed away. Using model life tables, these probabilities of death are transformed into age-specific death rate by location, year and sex. GBD has collated a large database of cause of death data from vital registrations and verbal autopsy surveys in which relatives are asked a standard set of questions to ascertain the likely cause of death, supplemented with police and mortuary data for injury deaths in countries with no other data. For countries with vital registration data, the completeness is assessed with demographic methods based on comparing recorded deaths with population counts between two successive censuses. The cause of death information is provided in a large number of different classification systems based on versions of the International Classification of Diseases or bespoke classifications in some countries. All data are mapped into the disease and injury categories of GBD. All classification systems contain codes that are less informative because they lack a

specific diagnosis (e.g. unspecified cancer) or refer to codes that cannot be underlying cause of death (e.g., low back pain or senility) or are intermediate causes (e.g., heart failure or sepsis). Such deaths are redistributed to more precise underlying causes of death.[4] After these redistributions and corrections for under-registration the data are analyzed in Code (cause of death ensemble model), a highly systematized tool that runs many different models on the same data and chooses an ensemble of models that best reflects all the available input data. Models are chosen with variations in the statistical approach (‘mixed effects’ of space-time Gaussian Process Regression), in the unit of analysis (rates or cause fractions), and the choice of predictive covariates. The statistical performance of all models is tested by holding out 30% of the data and checking how well a model covers the data that were held out. To enforce consistency from Code, the sum of all cause-specific mortality rates is scaled to that of the all-cause mortality rates in each age, sex, location and year category.

Non-fatal estimates are based on systematic reviews of published papers and unpublished documents, survey microdata, administrative records of health encounters, registries and disease surveillance systems. Our Global Health Data Exchange (God, http://ghdx.healthdata.org/) is the largest repository of health data globally. We first set a reference case definition and/or study method that best quantifies each disease or injury or consequence thereof. If there is evidence of a systematic bias in date that used different case definitions or methods compared to reference data we adjust those data points to reflect what its value would have been if measured as the reference. This is a necessary step if one wants to use all data pertaining to a particular quantity of interest rather than choosing a small subset of data of the highest quality only. Dismoded-MR 2.1, a Bayesian meta-regression tool, is our main method of analyzing non-fatal data. It is designed as a geographical cascade where a first model is run on all the world’s data which produces an initial global fit and estimates coefficients for predictor variables and the adjustments for alternative study characteristics. The global fit adjusted by the values of random effects for each of 7 GBD super-regions, the coefficients on sex and country predictors, are passed down as data to a model for each super-region together with the input data for that geography. The same steps are repeated going from super-region to 21 region fits and then to 195 fits by country and where applicable a further level down to subnational units. Below the global fit, all models are run separately by sex and for 6 time periods: 1990, 1995, 2000, 2005, 2010 and 2016. During each fit all data on prevalence, incidence, remission (i.e., cure rate) and mortality are forced to be internally consistent. For most diseases, the bulk of data on prevalence or incidence is at the disease level with fewer studies providing data on the proportions of cases of disease in each of the sequelae defined for the disease. The proportions in each sequela are pooled using Dismoded-MR 2.1 or meta-analysis or derived from analyses of patient-level data sets. The multiplication of prevalent cases for each disease sequela and the appropriate disability weight produces YLD estimates that do not yet take into account comorbidity. To correct for comorbidity, these data are used in a simulation to create hypothetical individuals in each age, sex, location and year combination who experience no, one or multiple sequelae simultaneously. We assume that disability weights are multiplicative rather than additive as this avoids assigning a combined disability weight value in any individual to exceed 1, i.e., be worse than a ‘year lost due to death’. This comorbidity adjustment leads to an average scaling down of disease-specific YLDs ranging from around 2% in young children up to 17% in oldest ages.

All our estimates of causes of death are categorical: each death is assigned to a single underlying cause. This has the attractive property that all estimates add to 100%. For risks, we use a different, ‘counterfactual’, approach, i.e. answering the question: what the burden would have been if the population had been exposed to a theoretical minimum level of exposure to a risk. Thus, we need to define what level of exposure to a risk factor leads to the lowest amount of disease. We then analyses data on the prevalence of exposure to a risk and derive relative risks for any risk-outcome pair for which we find sufficient evidence of a causal relationship. Prevalence of exposure is estimated in Dismoded-MR 2.1, using space-time Gaussian Process Regression, or from satellite imagery in the case of ambient air pollution. Relative risk data are pooled using meta-analysis of cohort, case-control and or intervention studies. For each risk and outcome pair, we evaluate the evidence and judge if the evidence falls into the categories of convincing or probable as defined by the World Cancer Research Fund.[5] From the prevalence and relative risk results, population attributable fractions are estimated relative to the theoretical minimum risk exposure level (TMREL). When we aggregate estimates for clusters of risks, e.g. metabolic or behavioral risks, we use a multiplicative function rather than simple addition and take into account how much of each risk is mediated through another risk. For instance, some of the risk of high body mass index is directly onto stroke as an outcome but much

of its impact if mediated through high blood pressure, high cholesterol or high fasting plasma glucose and we would not want to double count the mediated effects when we estimate aggregates across risk factors.[6]

Uncertainty is propagated throughout all these calculations by creating 1,000 values for each prevalence, death, YLL, YLD or DALY estimate and performing aggregations across causes and locations at the level of each of the 1,000 values for all intermediate steps in the calculation. The lower and upper bounds of the 95% uncertainty interval are the 25th and 975th values of the ordered 1,000 values.

GBD uses a composite indicator or sociodemographic development, SDI, which reflects the geometric mean of normalized values of a locations income per capita, the average years of schooling in the population 15 and over, and the total fertility rate. Countries and territories are grouped into five quintiles of high, high-middle, middle, low-middle, and low SDI based on their 2016 values.[2]

Detailed GBD 2016 results are available for download online at: **http://ghdx.healthdata.org/gbd-results-tool** (accessed July 19, 2018)

Appendix Figure 1. MS nonfatal health outcome estimation flow diagram

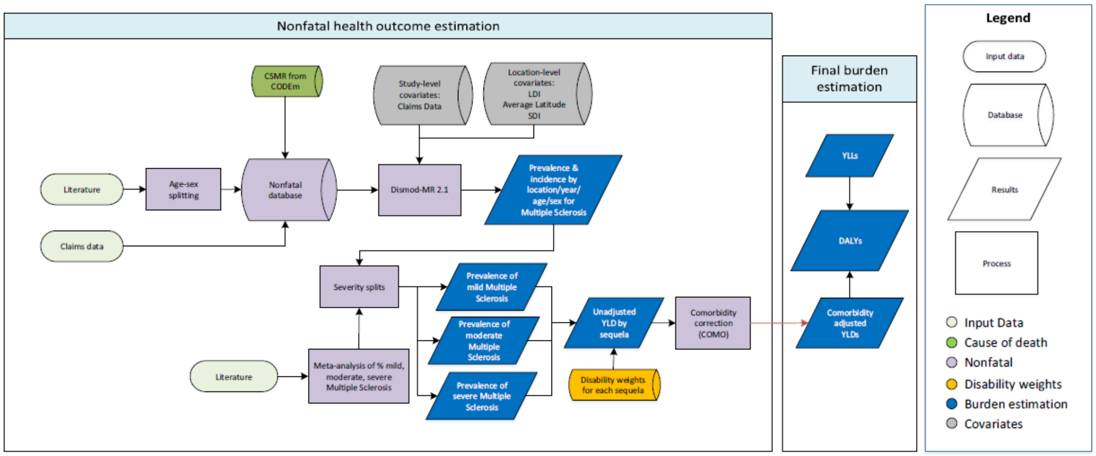

Case definition

Multiple sclerosis is a chronic, degenerative, and progressive neurological condition typified by the damaging of the myelin sheaths. For GBD, the McDonald's criteria for diagnosis are considered the gold standard, but other definitions such as Poser Committee's criteria and self-report of a doctor's diagnosis are also included. The ICD-10 code for MS is G35.

Input data

A systematic review was conducted for MS for GBD 2015. The search using (multiple sclerosis AND epidemiology AND ("2011/01/01"[PDat] : "2015/12/31"[PDat])) from 1/1/2011-7/15/15 yielded 1756 hits with 28 sources marked for extraction.

The data underpinning estimates of burden due to MS are generally of two types. The first are representative, population-based surveys. This includes retrospective case/hospital report analysis, nationally representative health studies and the like. Studies with no clearly defined sample or that draw from specific clinic/patient organizations were excluded during the systematic review phase. The second type are claims data from the United States from 2000, 2010, and 2012. Additional information on the source and preparation of these data is provided elsewhere.

The following table provides a description of the density and distribution of literature data informing the MS estimates

Appendix Table 1. Density and Distribution of the literature on multiple sclerosis

	Prevalence	Incidence	Mortality risk
Studies	132	65	14
Countries/subnationals	105	26	10
GBD world regions	11	8	3

Beyond the exclusion of studies using non-representative populations, there are no substantial adjustment or outlying criteria for the MS model. Certain studies have been outliered on a case-by-case basis due to: (1) subsequent review and exclusion due to inappropriate of the study design, and overly broad age and sex groups that conflict with existing gold standard age-sex specific data – where possible.

Severity splits

For GBD 2016, we updated the meta-analysis of all eligible studies that reported EDSS. The search using (("2008"[Date - Publication] : "2016"[Date - Publication])) AND (multiple sclerosis[MeSH Terms] OR multiple sclerosis) AND (epidemiology OR prevalence OR incidence) AND ("Kurtzke's Expanded Disability Status Scale") from 1/1/2008 to 11/14/2016 yielded 355 hits, with 10 marked for extraction.

As in GBD 2013, we use Kurtzke's Expanded Disability Status Scale (EDSS) to determine severity splits for MS. However, for GBD 2016 we added a category for asymptomatic multiple sclerosis in order to capture the initial stages of relapse-remitting multiple sclerosis which has no disability associated. The EDSS scores corresponding to each severity are as follows:

Asymptomatic: EDSS = 0
Mild: $0 < \text{EDSS} \leq 3.5$
Moderate: $3.5 < \text{EDSS} \leq 6.5$
Severe: $6.5 < \text{EDSS} \leq 9.5$

The table below illustrates severity levels, lay descriptions, and disability weights (DW).

Appendix Table 2. Multiple sclerosis severity level, lay descriptions and disability weight (DW) definitions

Severity level	Lay description	DW (95% CI)
Asymptomatic	-	0<br>(0-0)
Mild	Has mild loss of feeling in one hand, is a little unsteady while walking, has slight loss of vision in one eye, and often needs to urinate urgently.	0.183<br>(0.124–0.253)
Moderate	Needs help walking, has difficulty with writing and arm coordination, has loss of vision in one eye and cannot control urinating.	0.463<br>(0.313–0.613)
Severe	Has slurred speech and difficulty swallowing. The person has weak arms and hands, very limited and stiff leg movement, has loss of vision in both eyes and cannot control urinating.	0.719<br>(0.534–0.858)

Because not all sources had information on the number of cases with EDSS stage 0, instead reporting on a mild category, we implemented a two-step meta-analysis strategy. First, we subsetted the studies to those that reported

on the number of cases with EDSS stage 0, and did meta-analyses on the proportion of asymptomatic and mild cases. Then, we conducted meta-analyses on the full dataset to get the proportion mild, moderate and severe and we squeezed the asymptomatic and mild categories from the previous meta-analyses into the mild category established by the meta-analysis on the full dataset.

The following figures provide the result of the first meta-analysis on the asymptomatic and mild categories.

Appendix Figure 2. Asymptomatic cases of MS meta-analysis of studies

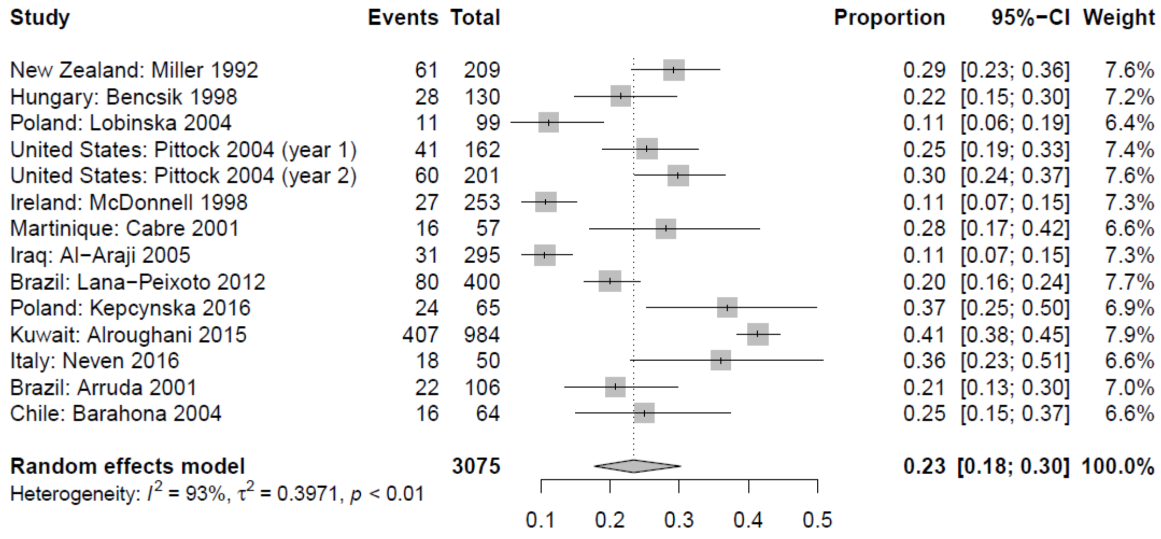

Appendix Figure 3. Mild cases of MS meta-analysis of studies

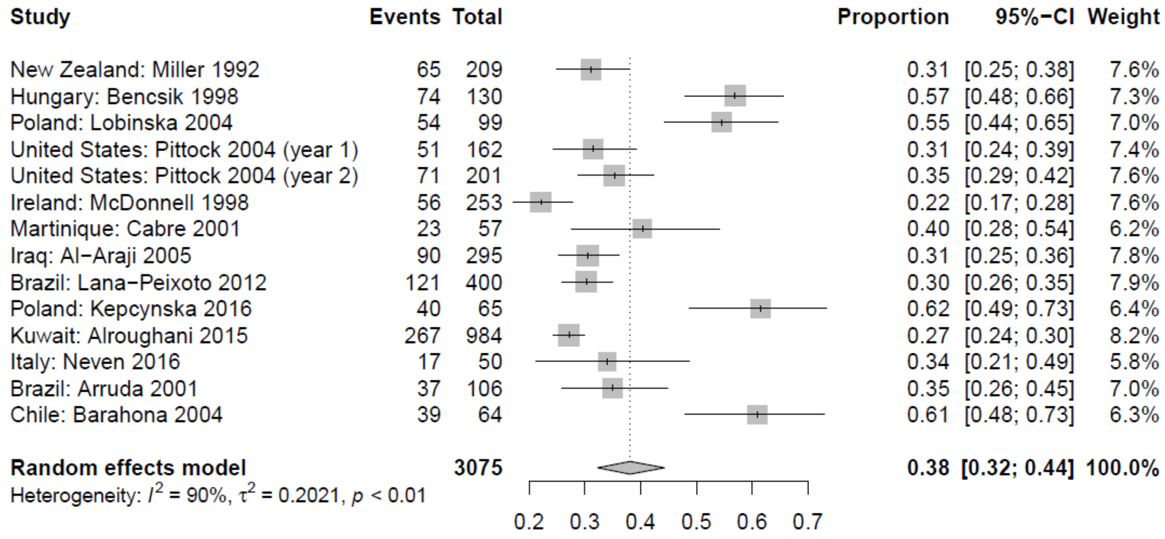

The following figures provide the result of the second meta-analysis on the mild, moderate and severe categories.

Appendix Figure 4. Mild Cases of MS (Including both Asymptomatic and Mild Categories) meta-analysis of studies

Study	Events	Total	Proportion	95%−CI	Weight
Italy: Casetta 1998	221	394	0.56	[0.51; 0.61]	4.1%
Japan: Houzen 2003	20	31	0.65	[0.45; 0.81]	3.1%
Hungary: Bencsik 2001	119	248	0.48	[0.42; 0.54]	4.1%
Spain: Arribas 1999	31	54	0.57	[0.43; 0.71]	3.5%
Spain: Pardo 1997	26	43	0.60	[0.44; 0.75]	3.4%
Taiwan: Tsai 2004	27	41	0.66	[0.49; 0.80]	3.3%
Brazil: da Silva 2016	84	208	0.40	[0.34; 0.47]	4.0%
Romania: Maier 2016	258	351	0.74	[0.69; 0.78]	4.1%
Iran: Torabipour 2014	282	332	0.85	[0.81; 0.89]	4.0%
Iran: Ayatollahi 2013	41	51	0.80	[0.67; 0.90]	3.2%
Netherlands: Karampampa 2013	121	263	0.46	[0.40; 0.52]	4.1%
Iran: Harandi 2012	47	78	0.60	[0.49; 0.71]	3.7%
France: Kobelt 2009	529	1334	0.40	[0.37; 0.42]	4.2%
New Zealand: Miller 1992	126	209	0.60	[0.53; 0.67]	4.0%
Hungary: Bencsik 1998	102	130	0.78	[0.70; 0.85]	3.8%
Poland: Lobinska 2004	65	99	0.66	[0.55; 0.75]	3.8%
United States: Pittock 2004 (year 1)	92	162	0.57	[0.49; 0.65]	4.0%
United States: Pittock 2004 (year 2)	131	201	0.65	[0.58; 0.72]	4.0%
Ireland: McDonnell 1998	83	253	0.33	[0.27; 0.39]	4.0%
Martinique: Cabre 2001	39	57	0.68	[0.55; 0.80]	3.5%
Iraq: Al−Araji 2005	121	295	0.41	[0.35; 0.47]	4.1%
Brazil: Lana−Peixoto 2012	201	400	0.50	[0.45; 0.55]	4.1%
Poland: Kepcynska 2016	64	65	0.98	[0.92; 1.00]	1.2%
Kuwait: Alroughani 2015	674	984	0.68	[0.65; 0.71]	4.2%
Italy: Neven 2016	35	50	0.70	[0.55; 0.82]	3.4%
Brazil: Arruda 2001	59	106	0.56	[0.46; 0.65]	3.9%
Chile: Barahona 2004	55	64	0.86	[0.75; 0.93]	3.2%
**Random effects model**		**6503**	**0.62**	**[0.56; 0.68]**	**100.0%**

Heterogeneity: $I^2 = 95\%$, $\tau^2 = 0.3961$, $p < 0.01$

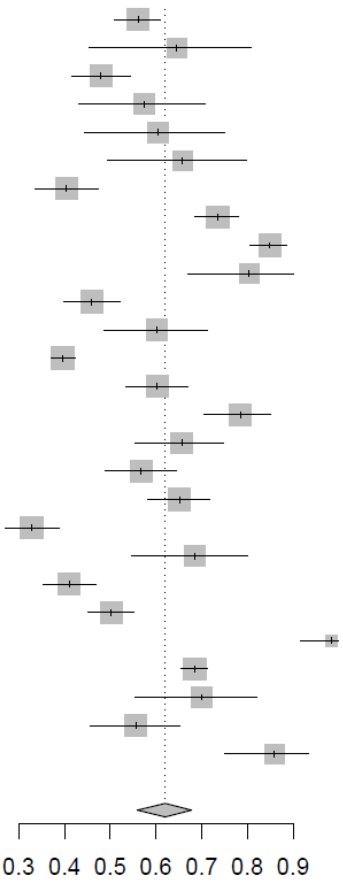

Appendix Figure 5. Moderate cases of MS meta-analysis of studies

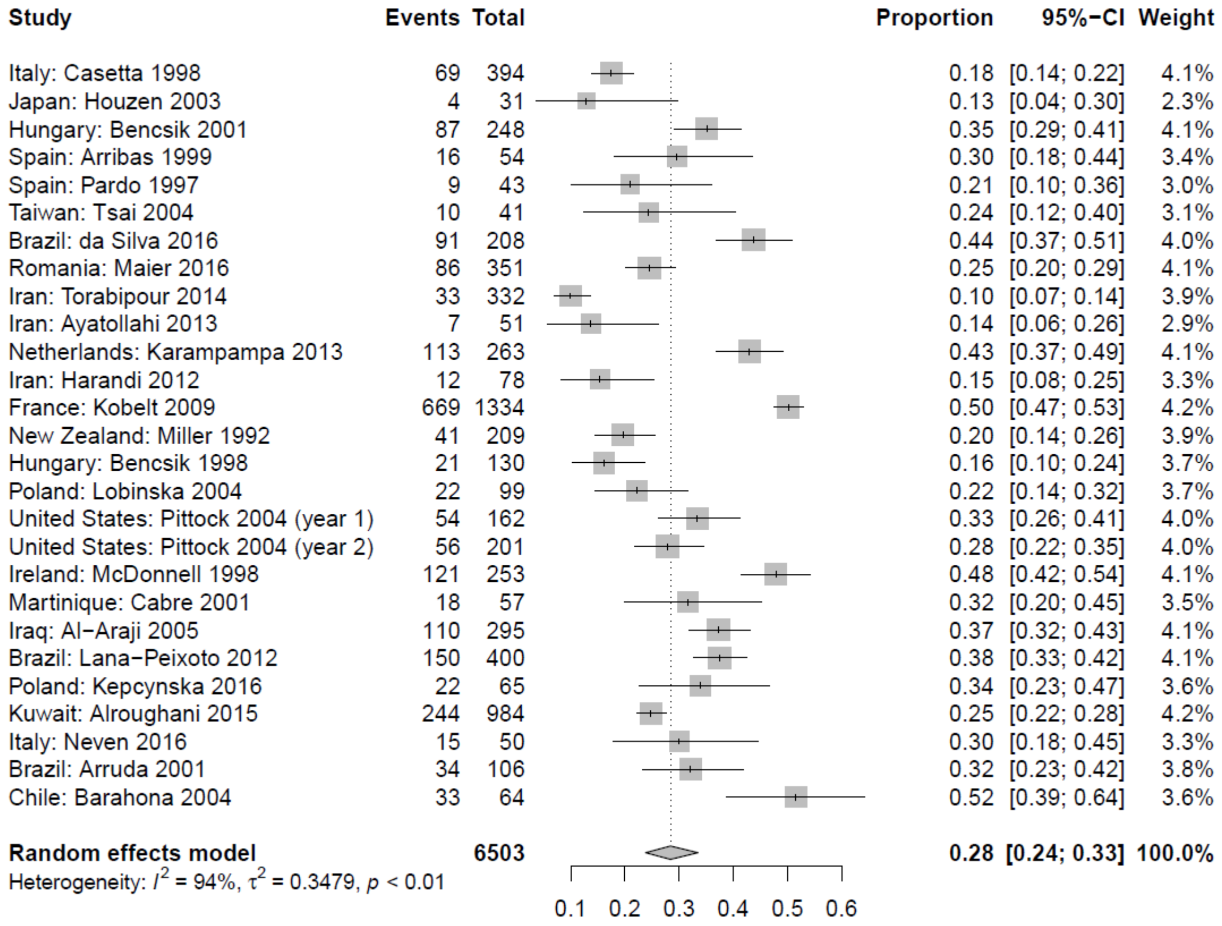

Appendix Figure 6. Severe Cases of MS meta-analysis of studies

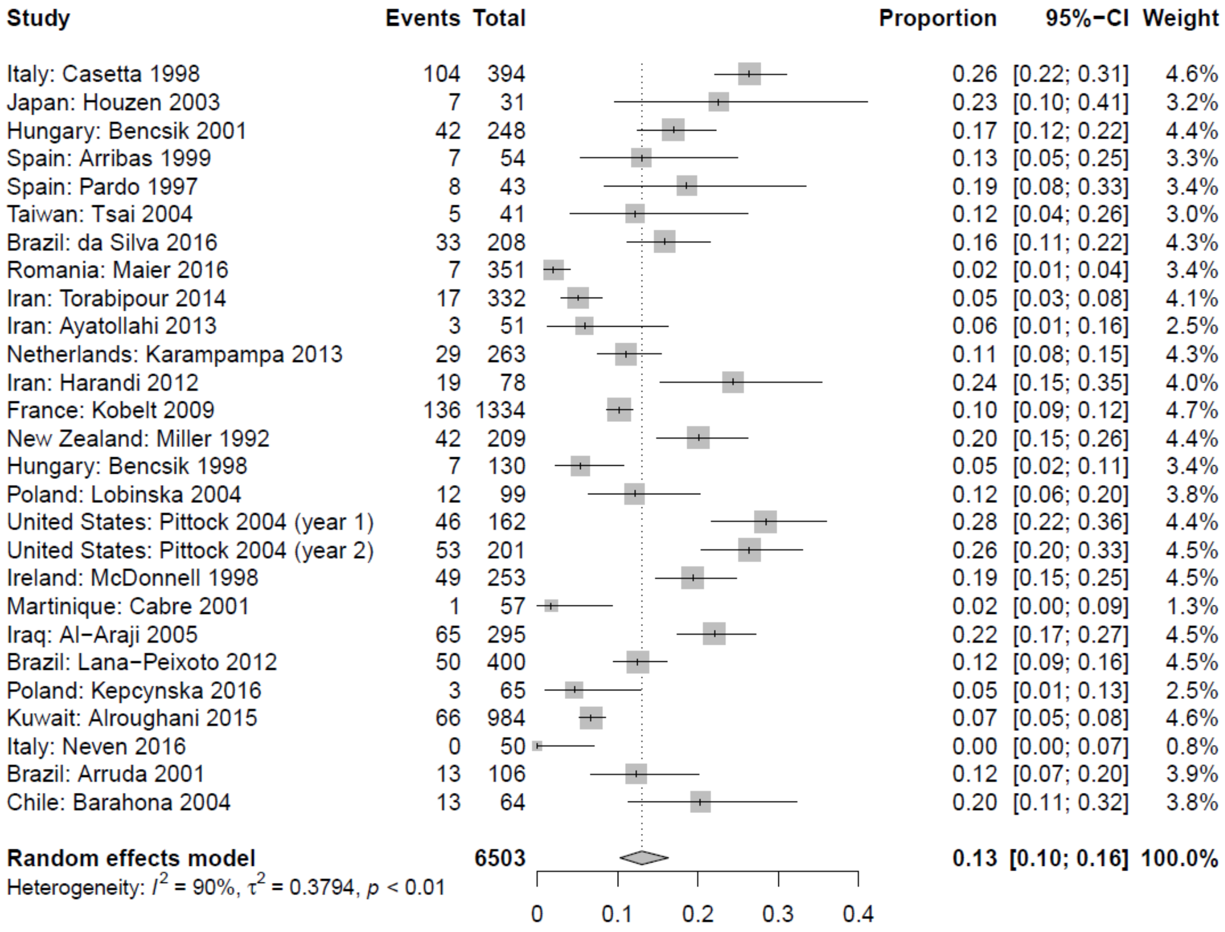

Modelling strategy

We use DisMod 2.1 as the main analytical tool for the MS estimation process. Prior settings include zero remission for all ages, and no incidence or excess mortality for persons under 4 years old. We also constrain the super-region random effects for prevalence and incidence to -0.5 and 0.5 to account for spurious inflation of regional differences.

Claims data for 2000 and 2010 are adjusted via study covariates to account for systematically low estimates relative to the 2012 claims data. Implicit in this adjustment is the assumption that variation between years of claims data is a function of data collection inconsistencies and noise.

Similar to other cases we use GBD estimates of cause-specific mortality rate (CSMR) and Excess Mortality Rate (EMR) in this model.

To assist the estimation process, we use a several country-level covariates. These effects plus those of the study covariates are presented in Appendix Table 3.

Appendix Table 3. Covariate definitions for the study of multiple sclerosis

Covariate	Measure	Beta	Exponentiated	Parameter Type
Absolute value of average latitude	prevalence	.029<br>(.026 - .040)	1.03<br>(1.03 - 1.04)	Country-level
Absolute value of average latitude	incidence	.055<br>(.0095 - .061)	1.06<br>(1.01 - 1.06)	Country-level
All MarketScan, year 2000	prevalence	-.26<br>(-.29 - -.22)	.77<br>(.75 - .80)	x-cov
All MarketScan, year 2010	prevalence	-.0011<br>(-.0034 - -.0017)	.99<br>(.97 – 1.00)	x-cov
Healthcare access and quality index	excess mortality rate	-.048<br>(-.21 - -.035)	.95<br>(.81 - .97)	Country-level
SDI	prevalence	1.98<br>(1.96- 2.00)	7.24<br>(7.06- 7.38)	Country-level

As described in the literature, extreme latitude is associated with higher prevalence and incidence of MS. While the pathway that affects MS is not fully understood, our results suggest a sizable relationship. Our operationalization of latitude is created by a population-weighted average of latitude by country and taking the absolute value. The underlying population distribution rasters are part of the Gridded Population of the World dataset.

Although there are no known cures for MS, we expect disease management to differ globally – largely as a function of available resources. To capture this, we use the healthcare access and quality index covariate to capture this relationship in the estimation of excess mortality.

To capture possible social and cultural risk factors or modifiers of MS prevalence, we include SDI as a covariate.

Appendix Figure 7. Cause of death estimation flow diagram

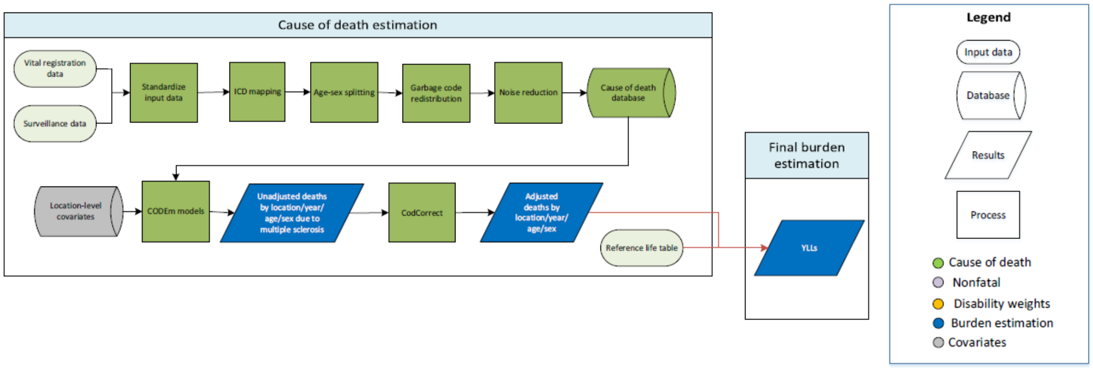

Input data

Data used to estimate multiple sclerosis included vital registration and surveillance data from the cause of death (COD) database. Our outlier criteria were to exclude data points that (1) were implausibly high or low, (2) substantially conflicted with established age or temporal patterns, or (3) significantly conflicted with other data sources conducted from the same locations or locations with similar characteristics (ie, Socio-demographic Index).

Modelling strategy

The standard CODEm modelling approach was used to estimate deaths due to multiple sclerosis. Separate models were conducted for male and female mortality, and the age range for both models was 20–95+ years. For GBD 2016, the health system access covariate was replaced by the new health care access and quality index covariate. Otherwise, there were no substantial changes from GBD 2015. The covariates used are displayed below.

Appendix Table 4. Covariates used for CODEm modelling for multiple sclerosis

Level	Covariate	Direction
1	absolute value of average latitude	+
2	animal fat consumption (kcal per capita)	+
	mean serum total cholesterol (mmol/L)	+
	health care access and quality index	-
3	cumulative cigarettes (10 years)	+
	cumulative cigarettes (5 years)	+
	education (years per capita)	-
	log-transformed LDI (per capita)	-
	smoking prevalence	+
	Socio-demographic Index	+

Appendix Table 5. Count of data sources used in nonfatal modeling for multiple sclerosis by 21 regions in 2016.

Region name	Incidence	Prevalence	Remission	Mortality	Claims data
**East Asia**	1	4	0	0	0
**Southeast Asia**	0	0	0	0	0
**Oceania**	0	0	0	0	0
**Central Asia**	1	0	0	0	0
**Central Europe**	5	12	0	0	0
**Eastern Europe**	0	0	0	0	0
**High-income Asia Pacific**	0	4	0	0	0
**Australasia**	2	4	0	1	0
**Western Europe**	45	71	0	10	0
**Southern Latin America**	1	0	0	0	0
**High-income North America**	3	11	0	3	3
**Caribbean**	0	0	0	0	0
**Andean Latin America**	0	0	0	0	0
**Central Latin America**	0	1	0	0	0
**Tropical Latin America**	0	3	0	0	0
**North Africa and Middle East**	7	16	0	0	0
**South Asia**	0	2	0	0	0
**Central sub-Saharan Africa**	0	0	0	0	0
**Eastern sub-Saharan Africa**	0	1	0	0	0
**Southern sub-Saharan Africa**	0	0	0	0	0
**Western sub-Saharan Africa**	0	0	0	0	0
	65	129	0	14	3

Appendix Table 6. GATHER checklist of information that should be included in reports of global health estimates, with description of compliance and location of information for GBD 2016.

#	GATHER checklist item	Description of compliance	Reference
**Objectives and funding**			
1	Define the indicators, populations, and time periods for which estimates were made.	Narrative provided in paper and appendix describing indicators, definitions, and populations	Main text (Methods) and appendix
2	List the funding sources for the work.	Funding sources listed in paper	Summary (Funding)
**Data Inputs**			
For all data inputs from multiple sources that are synthesised as part of the study:			
3	Describe how the data were identified and how the data were accessed.	Narrative description of data seeking methods provided	Main text (Methods) and appendix
4	Specify the inclusion and exclusion criteria. Identify all ad-hoc exclusions.	Narrative about inclusion and exclusion criteria by data type provided; ad hoc exclusions in cause-specific write-ups	Main text (Methods) and appendix
5	Provide information on all included data sources and their main characteristics. For each data source used, report reference information or contact name/institution, population represented, data collection method, year(s) of data collection, sex and age range, diagnostic criteria or measurement method, and sample size, as relevant.	An interactive, online data source tool that provides metadata for data sources by component, geography, cause, risk, or impairment has been developed	Online data citation tools: http://ghdx.healthdata.org/gbd-2016
6	Identify and describe any categories of input data that have potentially important biases (e.g., based on characteristics listed in item 5).	Summary of known biases by cause included in appendix	Appendix
For data inputs that contribute to the analysis but were not synthesised as part of the study:			
7	Describe and give sources for any other data inputs.	Included in online data source tool	http://ghdx.healthdata.org/gbd-2016
For all data inputs:			
8	Provide all data inputs in a file format from which data can be efficiently extracted (e.g., a spreadsheet as opposed to a PDF), including all relevant meta-data listed in item 5. For any data inputs that cannot be shared due to ethical or legal reasons, such as third-party ownership, provide a contact name or the name of the institution that retains the right to the data.	Downloads of input data available through online tools, including data visualisation tools and data query tools; input data not available in tools will be made available upon request	Online data visualisation tools, data query tools, and the Global Health Data Exchange
**Data analysis**			
9	Provide a conceptual overview of the data analysis method. A diagram may be helpful.	Flow diagrams of the overall methodological processes, as well as cause-specific modelling processes, have been provided	Main text (Methods) and appendix
10	Provide a detailed description of all steps of the analysis, including mathematical formulae. This description should cover, as relevant, data cleaning, data pre-processing, data adjustments and weighting of data sources, and mathematical or statistical model(s).	Flow diagrams and corresponding methodological write-ups for each cause, as well as the databases and modelling processes, have been provided	Main text (Methods) and appendix
11	Describe how candidate models were evaluated and how the final model(s) were selected.	Provided in the methodological write-ups	Appendix
12	Provide the results of an evaluation of model performance, if done, as well as the results of any relevant sensitivity analysis.	Provided in the methodological write-ups	Appendix
13	Describe methods for calculating uncertainty of the estimates. State which sources of uncertainty were, and were not, accounted for in the uncertainty analysis.	Appendix	Appendix
14	State how analytic or statistical source code used to generate estimates can be accessed.	Appendix	http://ghdx.healthdata.org/gbd-2016-code
**Results and Discussion**			
15	Provide published estimates in a file format from which data can be efficiently extracted.	GBD 2016 results are available through online data visualisation tools, the Global Health Data	Main text, and online data tools

		Exchange, and the online data query tool	(data visualisation tools, data query tools, and the Global Health Data Exchange)
16	Report a quantitative measure of the uncertainty of the estimates (e.g. uncertainty intervals).	Uncertainty intervals are provided with all results	Main text, appendix, and online data tools (data visualisation tools, data query tools, and the Global Health Data Exchange)
17	Interpret results in light of existing evidence. If updating a previous set of estimates, describe the reasons for changes in estimates.	Discussion of methodological changes between GBD rounds provided in the narrative of the manuscript and appendix	Main text (Methods and Discussion) and appendix
18	Discuss limitations of the estimates. Include a discussion of any modelling assumptions or data limitations that affect interpretation of the estimates.	Discussion of limitations provided in the narrative of the main paper, as well as in the methodological write-ups in the appendix	Main text (Limitations) and appendix

Appendix Figure 8. Number of datapoints used for MS morbidity and mortality estimates for each country. The gray shading represents no data points available for these countries for the period 1980-2016. A study can contribute multiple data points, e.g. for a specific age group or gender.

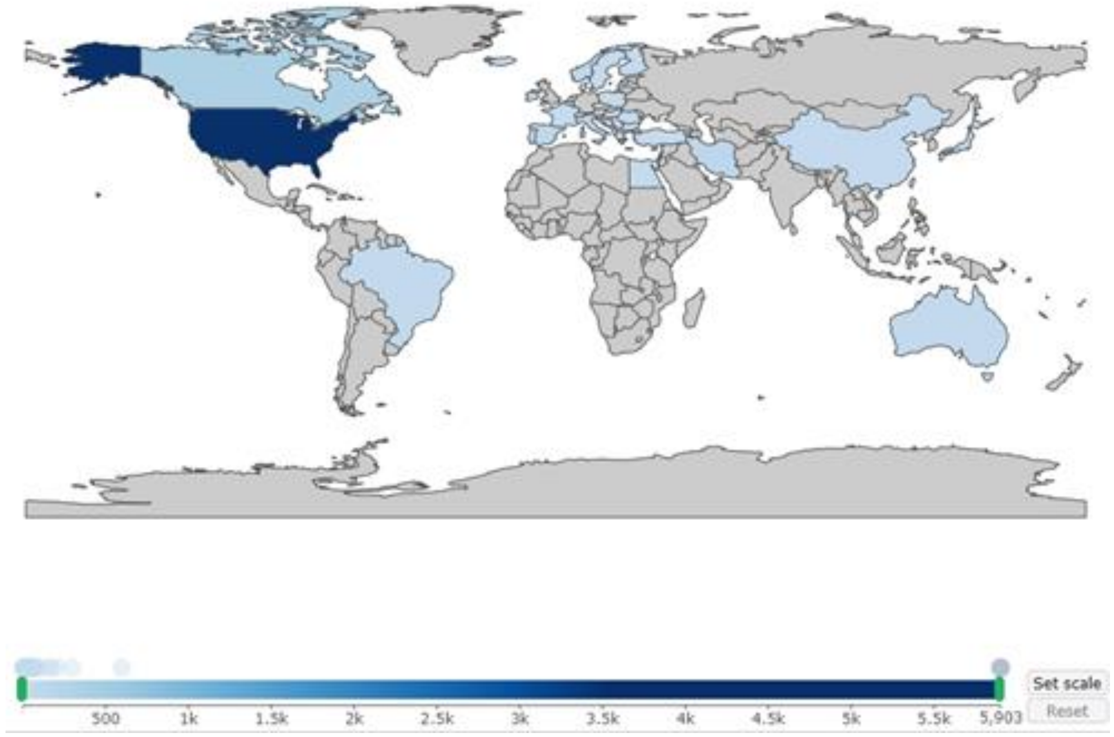

References:

1. GBD 2015 Neurological Disorders Collaborator Group. Global, regional, and national burden of neurological disorders during 1990-2015: a systematic analysis for the Global Burden of Disease Study 2015. *Lancet* Neurol 2017; **16:877**–97.

2. GBD 2016 Mortality Collaborators. Global, regional, and national under-5 mortality, adult mortality, age-specific mortality, and life expectancy, 1970-2016: a systematic analysis for the Global Burden of Disease Study 2016. *Lancet* 2017; **390:1084**–150.

3. Salomon JA, Hageman JA, Davis A, et al. Disability weights for the Global Burden of Disease 2013 study. *Lancet Glob Health* 2015;**3**: e712-723.

4. GBD 2016 Causes of Death Collaborators. Global, regional, and national age-sex specific mortality for 264 causes of death, 1980-2016: a systematic analysis for the Global Burden of Disease Study 2016. *Lancet* 2017; **390**:1151–210.

5. American Institute for Cancer Research. Food, nutrition, physical activity, and the prevention of cancer: a global perspective. Washington, DC: American Institute for Cancer Research, 2007.

6. GBD 2016 Risk Factors Collaborators. Global, regional, and national comparative risk assessment of 84 behavioural, environmental and occupational, and metabolic risks or clusters of risks, 1990-2016: a systematic analysis for the Global Burden of Disease Study 2016. *Lancet* 2017; **390**: 1345–422.

7. GBD 2013 Mortality and Causes of Death Collaborators. Global, regional, and national age-sex specific all-cause and cause-specific mortality for 240 causes of death, 1990-2013: a systematic analysis for the Global Burden of Disease Study 2013. *Lancet* 2015; **385**: 117–71.

8. United Nations Department of Economics and Social Affairs Population Division. World Population Prospects: The 2012 Revision. http://esa.un.org/unpd/wpp/Documentation/publications.htm (accessed July 19, 2018).
